# Local environment drives rapid shifts in composition and phylogenetic clustering of seagrass microbiomes

**DOI:** 10.1038/s41598-023-30194-x

**Published:** 2023-03-04

**Authors:** Melissa R. Kardish, John. J. Stachowicz

**Affiliations:** 1 Department of Evolution and Ecology, University of California, One Shields Avenue, Davis, CA 95616 USA; 2 Center for Population Biology, University of California, One Shields Avenue, Davis, CA 95616 USA

**Keywords:** Community ecology, Microbial ecology, Marine biology

## Abstract

Plant microbiomes depend on environmental conditions, stochasticity, host species, and genotype identity. Eelgrass (*Zostera marina)* is a unique system for plant–microbe interactions as a marine angiosperm growing in a physiologically-challenging environment with anoxic sediment, periodic exposure to air at low tide, and fluctuations in water clarity and flow. We tested the influence of host origin versus environment on eelgrass microbiome composition by transplanting 768 plants among four sites within Bodega Harbor, CA. Over three months following transplantation, we sampled microbial communities monthly on leaves and roots and sequenced the V4–V5 region of the 16S rRNA gene to assess community composition. The main driver of leaf and root microbiome composition was destination site; more modest effects of host origin site did not last longer than one month. Community phylogenetic analyses suggested that environmental filtering structures these communities, but the strength and nature of this filtering varies among sites and over time and roots and leaves show opposing gradients in clustering along a temperature gradient. We demonstrate that local environmental differences create rapid shifts in associated microbial community composition with potential functional implications for rapid host acclimation under shifting environmental conditions.

## Introduction

Host-associated microbial communities have important effects on their host^1,2^. The composition and structure of host-associated microbial communities vary by geographic region^3,4^, host health^5^, successional stage^6–9^ and environmental conditions^10,11^, but the extent to which the host is a cause of, or faces consequences from, this variation is often unclear^12^. Making progress on this question requires an understanding of the forces structuring these communities and is an important first step toward developing manipulative approaches that can rigorously test the composition and function of the host microbiome under environmental change. This requires experimental and observational work to disentangle the roles of temporal and environmental variation in driving community structure at different scales.

Microbial communities, like many ecological communities, vary with seasonal and interannual variation in environmental drivers and host characteristics^7,13,14^. However, whether these changes are due to direct environmental changes or changes in the host is often unclear. For example, corals transplanted among sites had bacterial communities characteristic of their destination rather than origin after 21 months^15^. Similarly, temporal/seasonal variation was the dominant driver of sponge microbiome composition among nearby sites, despite differences in water depth^13^. Some terrestrial plant–microbe common garden studies that have run for at least two years also find a dominance of local environmental over host characteristics in driving microbial assemblages^16^. However, the coarse temporal resolution of these studies limits the ability to assess how quickly a change in local environment changes microbiomes. Thus, the speed of microbiomes matching novel environments is often unknown, yet is critical for assessing the capacity of the microbiome to buffer the host against a changing environment.

Community composition is determined by the source pool of potential inhabitants in conjunction with numerous abiotic and biotic filters restricting certain members^17,18^. In order to assess the various roles of different filters, community ecologists have used functional similarity to describe likely niche overlap between members of a guild: using the observed patterns in communities to infer the process of assembly^19^. When good functional data is not available, as is the case for many uncultured members of metagenomic sequenced communities, phylogenetic similarity can be used, with the assumption that conservation of key traits is held within clades^20–23^. While this assumption does not always hold in microbial communities^24^, in many microbial groups this is a reasonable proxy for at least some key metabolic traits which appear conserved at a level correlated to 16S rRNA gene sequences^25,26^. Given this assumption, if microbial communities are phylogenetically clustered (i.e., more phylogenetically similar than expected by chance), local environmental filtering with respect to these conserved traits may exert a dominant influence on microbiomes. Alternatively, if microbiomes are phylogenetically overdispersed (less similar than expected by chance) resource partitioning or inter-taxon facilitation (i.e., cross-feeding, environmental buffering) could be the dominant driver of assembly^20^. At a minimum, variation in phylogenetic clustering vs dispersion in space or time or between different host tissues can generate hypotheses about the conditions under which different drivers influence assembly of microbiomes that can be tested in future studies.

Microbial communities associated with the marine angiosperm eelgrass (*Zostera marina*) are of increasing interest for their role in host ecology and ecosystem functioning. Eelgrass is a foundation species that provides food and habitat for a diversity of animals^27^, stabilizes sediment^28,29^, and mediates nutrient cycling^30^. The potential role of the microbiome in mediating these effects will clarify how to best conserve and restore the important functions of eelgrass. Both leaves and roots differ in composition from source pools in water and sediments, but roots are more differentiated from sediment than leaves are from water^31^. Both leaves and roots show geographic structure^31,32^, though this is more limited in roots than leaves. Potentially functionally important sulfide oxidizing bacteria are widespread and abundant in communities collected from roots^33^, and there is association between leaf microbial taxa including cell-wall degrading bacteria and the abundance of pathogens^34^ and disease severity^35^. No work in eelgrass to date has tested the role of host versus environment in microbial community structure but we know that there are strong differences in host genotypic composition, genetically-based traits, and environment on spatial scales less than 1km^36–39^ and that the plant microbiome varies on local, regional and global scales^31,35^.

**Figure 1:**
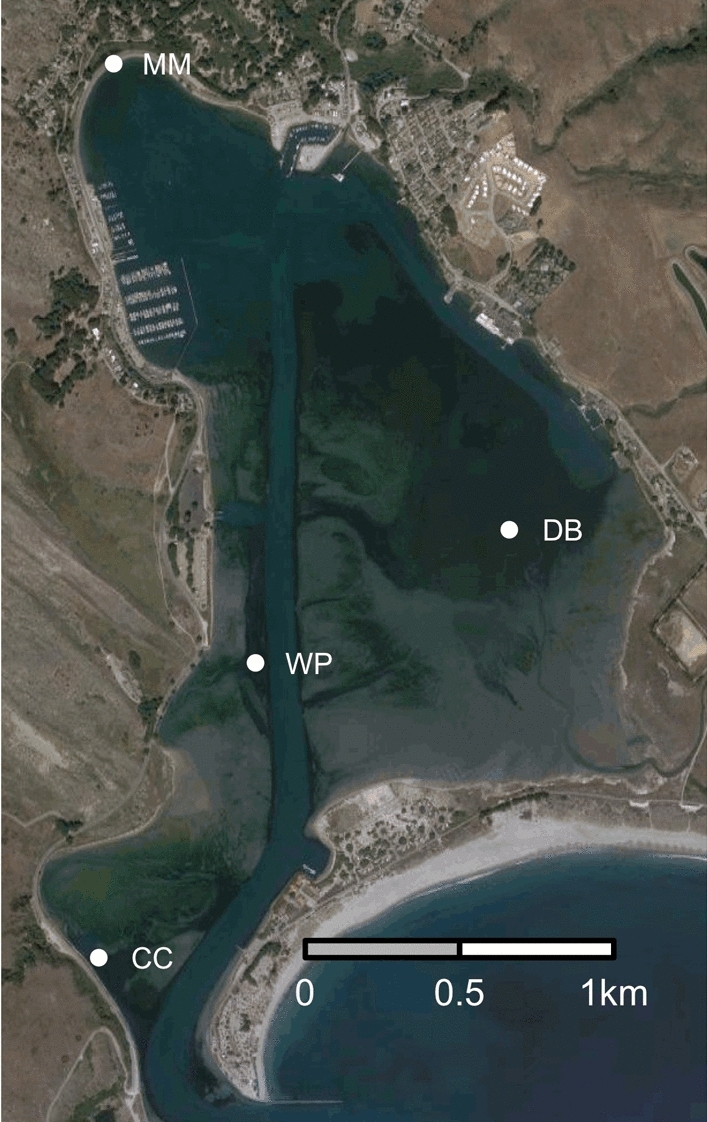
Map of reciprocal transplant sites in Bodega Harbor, Bodega Bay, CA. Campbell Cove (CC) is the site closest to the mouth of the harbor and a mean temperature of 15.8 ˚C during our experiment. Doran Beach (DB) is the least impacted by human activities in the harbor (farther from clamming) and had a mean temperature of 15.9 ˚C during our experiment. Mason’s Marina (MM) has finer grained sediment than other sites and is a restored site with patchier seagrass growth; in other experiments its temperature profile has been intermediate between the cooler sites and WP (HOBO logger at this site was lost). Westside Park (WP) had a mean temperature of 16.3 ˚C during our experiment and is used for many seagrass experiments within Bodega Harbor. We generated this map from the Google Maps Static API version 2 (maps.googleapis.com) using the R package “ggmap”.^74^.

To understand the assembly processes of seagrass microbiomes, we reciprocally transplanted eelgrass plants among four sites within a single 5 km^2^ embayment in Bodega Harbor, California, USA. These sites occur along a gradient from the mouth of the harbor (most oceanic) to the head (Fig. 1) that includes increasing temperature, decreasing water flow, decreasing sediment grain size and increasing sediment organic content, all of which potentially influence the root and leaf microbiomes. Local adaptation of plants to these differences could also moderate microbiome assembly. We also assessed the extent to which the experimental procedure: the process of uprooting, handling, transport and lab processing affected root and leaf microbiomes by comparing the bacterial communities from plants transplanted back to their origin site with undisturbed plants. We tracked changes in the bacterial assemblages associated with eelgrass roots and leaves across a time course of three months (July to September) to examine the relative importance of host factors vs environment in determining microbiome composition. We then applied community phylogenetic approaches to assess the role of environmental filtering vs. other processes in structuring bacterial assemblages.

## Results

### Sampling and sequencing success and results

We identified 43,118 bacterial ASVs across 681 leaf and root samples after quality filtering samples to 15,102,767 reads. Root samples contained between 239 and 1167 bacterial ASVs on their surface (we measured 330 root samples with read depth between 5981 and 80,762 reads), and leaf samples between 118 and 1010 bacterial ASVs (307 leaf samples with between 1980 reads and 65,621 per sample).

### On leaves, microbial communities strongly resembled destination and not origin site after one month

**Figure 2:**
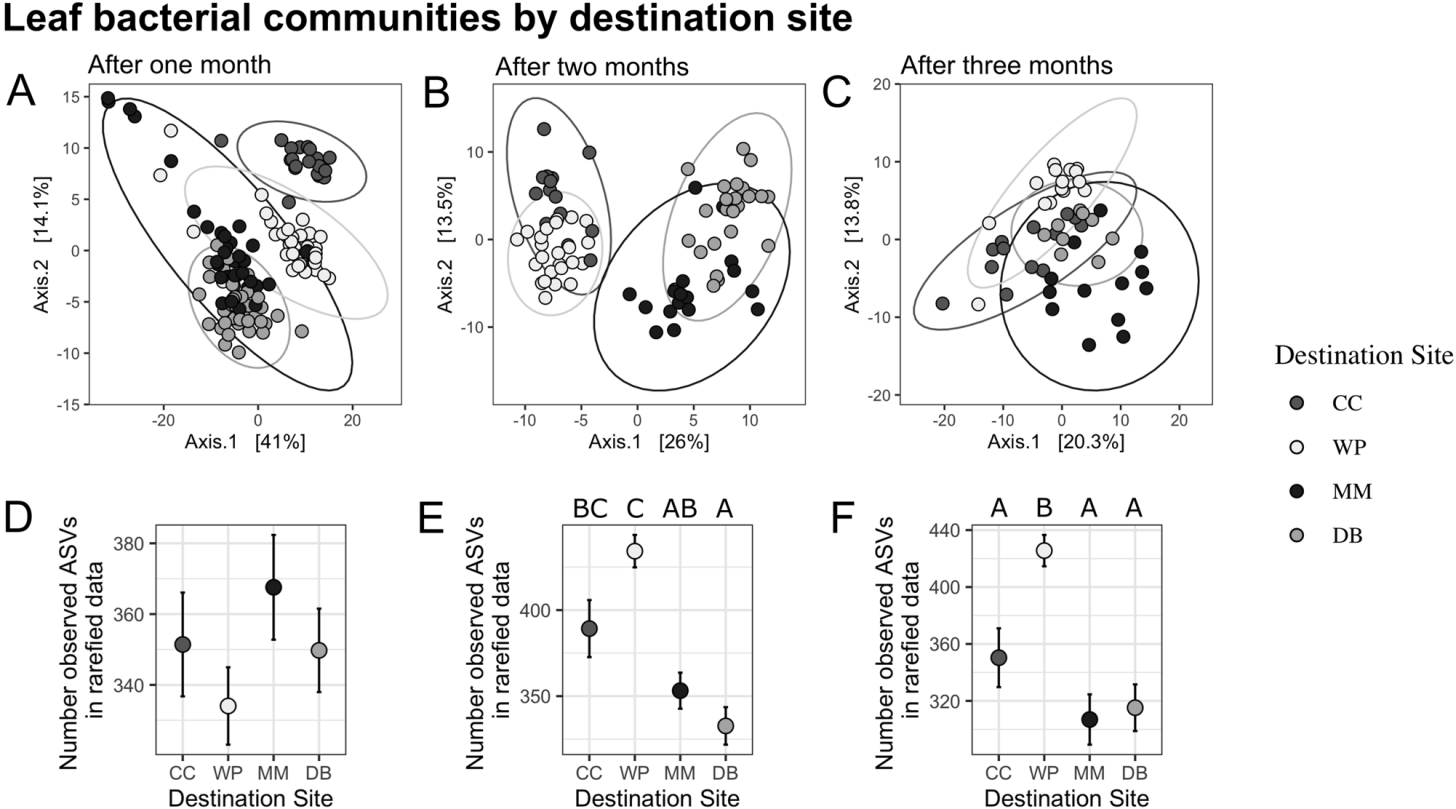
Differences in leaf microbial communities among destination sites. (**A**–**C**) Ordination of leaf bacterial community structure based on principal coordinate analysis of phylogenetic-isometric log-ratio transformed distances showing differences among sites by destination site (**A**) after one month, (**B**) after two months, and (**C**) after 3 months. All destination sites were distinct from others at all three timepoints (see Table 1). (**D**–**F**) Mean (+ /− 1 SE) amplicon sequence variant (ASV) richness on leaves at each site after 1, 2, and 3 months. Different letters above data points indicate significantly different ASV richness among destination sites.

Leaf bacterial community composition based on phylogenetic isometric log-ratio transformed data from samples transplanted among our four sites quickly resembled destination site (the site to which shoots were transplanted) and not origin site (the site of collection) across our four sites (Fig. 2, Table 1), suggesting that eelgrass leaf microbiomes rapidly change as a function of local conditions.

**Table 1:** Results of PERMANOVA indicating differences among transplanted leaf microbial communities (see Fig. 2).

		**df**	**Sum Of Squares**	**R**^**2**^	**F-Statistic**	**Pr(> F)**
After one month						
	Destination Site	3	9433.009	0.312	20.302	**0.001**
	Origin Site	3	1050.953	0.035	2.262	**0.009**
	Destination : Origin	7	1760.358	0.058	1.624	**0.015**
	Residual	116	17,966.060	0.595	NA	NA
	Total	129	30,210.379	1	NA	NA
After two months						
	Destination Site	3	6100.417	0.371	14.782	**0.001**
	Origin Site	3	511.728	0.031	1.240	0.174
	Destination : Origin	9	1157.904	0.070	0.935	0.66
	Residual	63	8666.808	0.527	NA	NA
	Total	78	16,436.857	1	NA	NA
After three months						
	Destination Site	3	3759.551	0.289	6.242	**0.001**
	Origin Site	3	652.923	0.050	1.084	0.301
	Destination : Origin	8	1573.425	0.121	0.980	0.547
	Residual	35	7026.486	0.540	NA	NA
	Total	49	13,012.385	1	NA	NA

Significant values are in bold.

**Figure 3:**
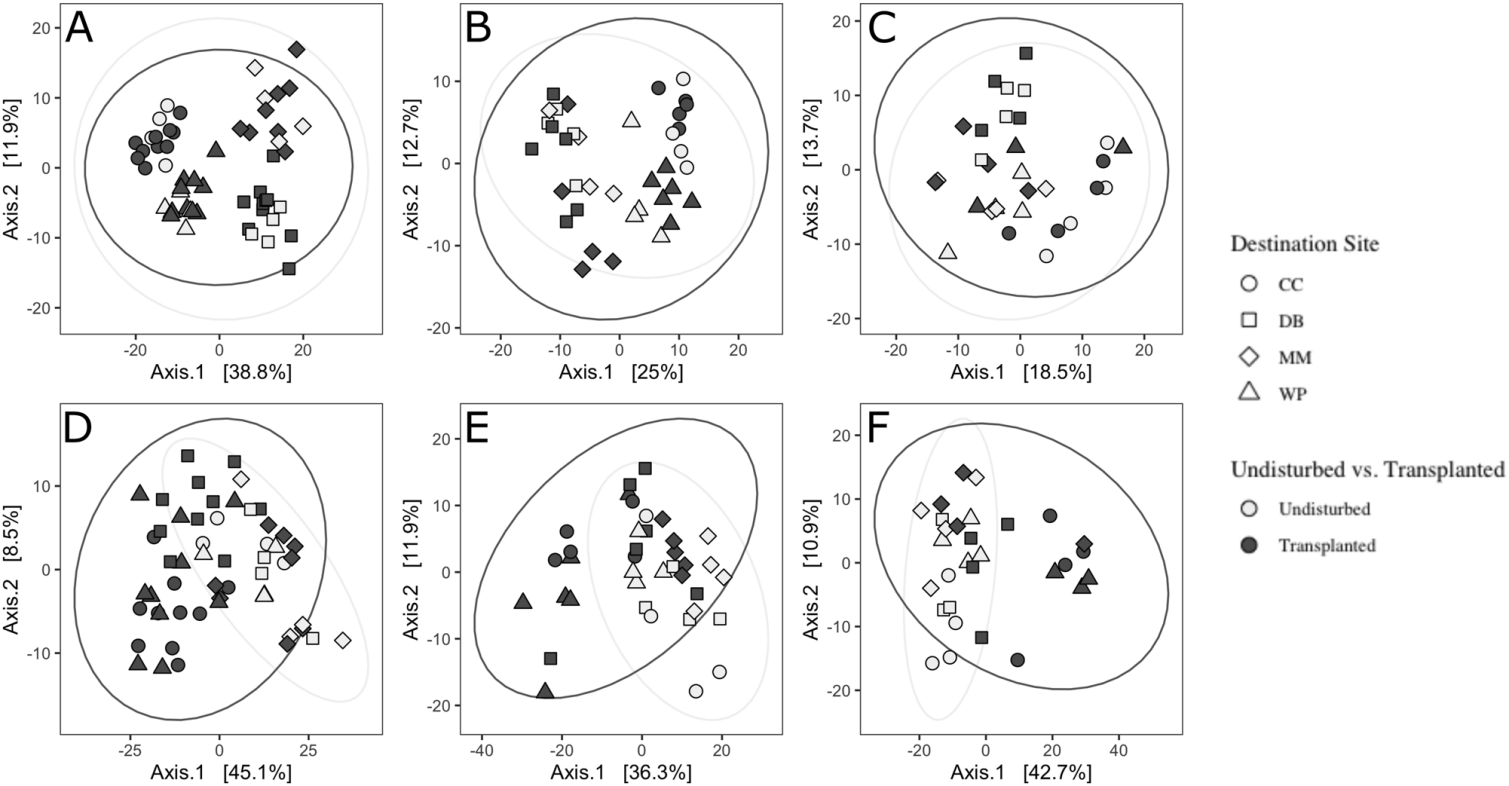
Differences in microbiome between home site transplants and transplant controls. Ordination of leaf bacterial community structure based on principal coordinate analysis of phylogenetic-isometric log-ratio transformed distances after (**A**) one month, (**B**) two months, and (**C**) three months. (**D**–**F**) Ordination of root bacterial community structure based on principal coordinate analysis of phylogenetic-isometric log-ratio transformed distances after (**D**) one month, (**E**) two months, and (**F**) three months. Leaf microbiomes were indistinguishable between transplant controls and undisturbed plants at any time point (**A**–**C**) and retained the among-site differences described in Fig. 2. Root microbiomes were distinct between transplant controls and undisturbed plants at all time points (**D**–**F**), while both transplanted and undisturbed root microbiomes demonstrated differences among sites.

After 1 month, there was also an effect of origin site and its interaction with destination site, though these effects were weaker than the effect of destination site (Fig. 2A, PERMANOVA results in Table 1). After two and three months, there was still a strong effect of destination site, but no effect of origin site or its interaction with destination site (Fig. 2B,C, Table 1). There was strong differentiation by destination site across all three time points (Fig. 2A–C, Table 1). Leaf microbiomes were indistinguishable between transplant controls and undisturbed plants at any time point (Table 2, Fig. 3A–C), and retained the among-site differences described in Fig. 2. This suggests that destination environment, and/or plant changes that happen as a result of the destination environment, were the primary drivers of leaf microbiome and these overwhelmed any residual effect of origin site within 2 months at most. These differences were associated with differences in variance among sites within time points (betadisper, ANOVA *p*_1_ = 0.0016, *p*_2_ = 0.0003, *p*_3_ = 0.05127): after one month, Mason’s Marina (MM) had higher within-site variance than all other sites, and after two months was still greater than Doran Beach (DB) and Westside Park (WP) but not Campbell Cove (CC). This implies that compositional differences at destination sites might not be different at MM than other sites in leaves, but might only be driven by an increase in variance.

**Table 2:** Results of PERMANOVA showing differences among based on variation in transplant status in leaves (Fig. 3A-C).

		**df**	**Sum Of Squares**	**R**^**2**^	**F-Statistic**	**Pr(> F)**
After one month						
	Site	3	10,979.408	0.511	18.072	**0.001**
	Transplant Status	1	370.765	0.017	1.831	0.091
	Destination Site:Transplant Status	3	819.616	0.038	1.349	0.145
	Residual	46	9315.596	0.434	NA	NA
	Total	53	21,485.385	1	NA	NA
After two months						
	Site	3	4093.618	0.349	6.359	**0.001**
	Transplant Status	1	282.124	0.024	1.315	0.158
	Site:Transplant Status	3	930.523	0.079	1.446	0.052
	Residual	30	6437.063	0.548	NA	NA
	Total	37	11,743.328	1	NA	NA
After three months						
	Site	3	2938.888	0.279	3.559	**0.001**
	Transplant Status	1	413.074	0.039	1.501	0.08
	Site:Transplant Status	3	848.899	0.081	1.028	0.407
	Residual	23	6331.219	0.601	NA	NA
	Total	30	10,532.080	1	NA	NA

Significant values are in bold.

As there was a significant interaction between destination and origin sites after one month, we compared all possible combinations of origin and destination sites. Regardless of origin site, bacterial communities differed between all pairwise destination sites after one month (Supplemental Tables S1 and S2 for *p* and R^2^ values). After one month, leaves of plants transplanted to DB from MM were distinct from those transplanted from CC or DB. Leaf bacterial communities of plants transplanted to MM from CC and MM were also still distinct from each other (Supplemental Tables S3 and S4 for pairwise *p* and R^2^ values). All pairwise combinations of leaf bacterial communities after two and three months were distinct from each other by destination site only.

Alpha diversity also varied as a function of destination site, although these differences only emerged after 2 months. After one month, there were no significant differences in amplicon sequence variant (ASV) richness among destination or origin sites (negative binomial GLM, *p* > 0.05, Fig. 2D). After two months, plants at CC had a higher number of ASVs than those at DB and plants at WP had a higher number of ASVs observed than MM and DB (negative binomial GLM, * p* < 0.001, all significant pairwise comparisons also *p* < 0.001; Fig. 2E). Finally, after three months, WP had a higher number of ASVs than DB and MM (*p*_vsDB_ < 0.001, *p*_vsMM_ < 0.001) and CC had more ASVs than DB (*p* < 0.001, Fig. 2F). After two months, there was also a small effect of origin site on richness due to differences between plants from CC and MM (*p* = 0.040). There were no differences in alpha diversity or variance between transplant leaf bacterial communities and untransplanted controls at any timepoint, except after 3 months, when transplanted plants had slightly higher bacterial richness than controls (ANOVA negative binomial GLM, *p*_1_ = 0.942, *p*_2_ = 0.942, *p*_3_ = 0.036; Supplemental Fig. S8A–C). Considering all time points, fewer ASVs were present on leaves when at DB as destination site and more bacterial ASVs on leaves when at WP.

### Root microbial communities similarly strongly resembled destination and not origin site after one month

**Figure 4:**
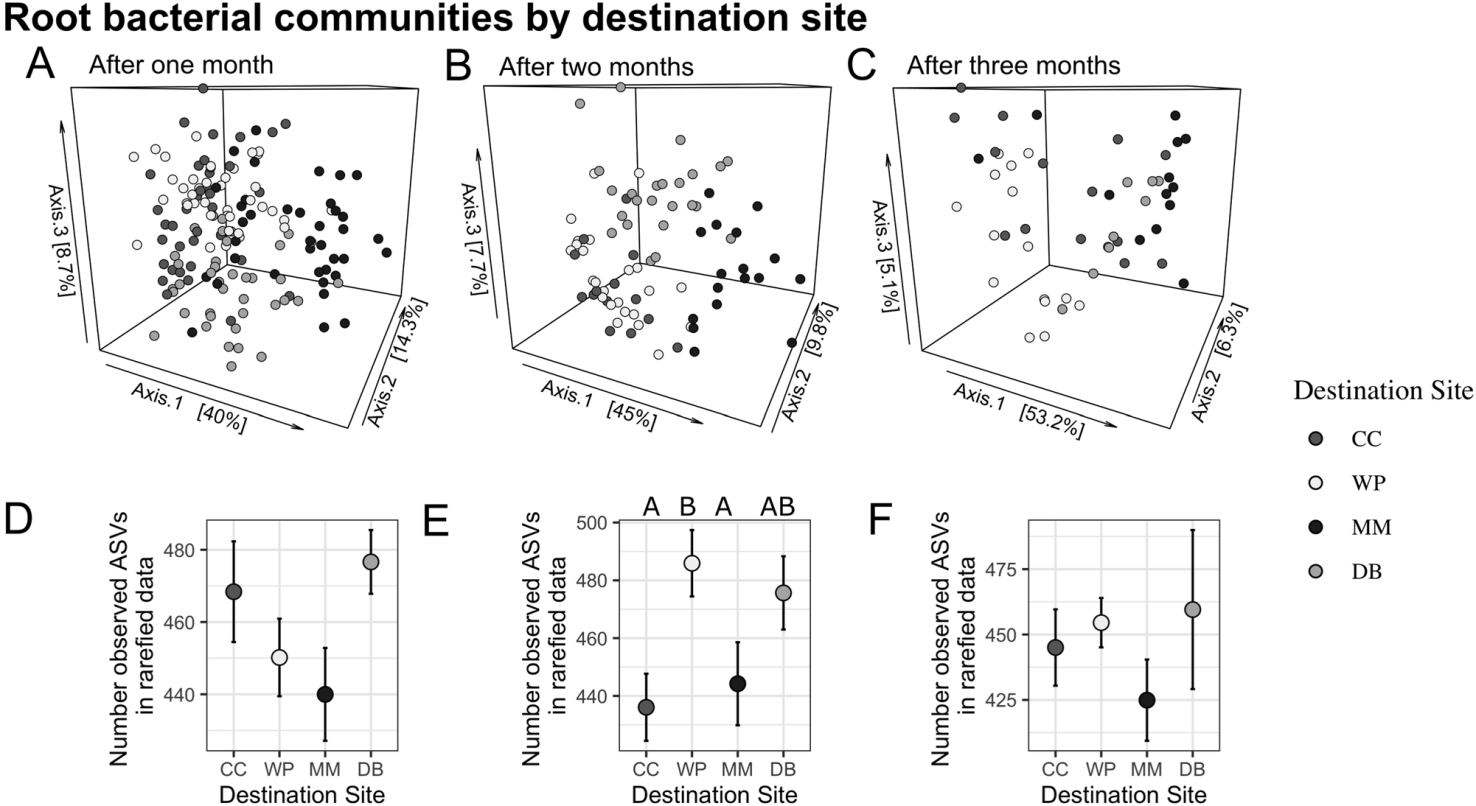
Differences in root microbial communities among destination sites. (**A**–**C**) 3-D ordination of root bacterial community structure based on principal coordinate analysis of phylogenetic-isometric log-ratio transformed distances. (**A**) shows differences among sites by destination site after one month, (**B**) after two months, and (C) after 3 months. All destination sites were distinct from others at all three timepoints (see Table 2). (D-F) Mean amplicon sequence variant (ASV) richness on roots at each site at 1, 2, and 3 months. Different letters above data points indicate significantly different ASV richness among destination sites.

Root bacterial communities also were most strongly influenced by destination site, though results were slightly more complex than for leaves. After one month, destination site had the strongest effect on microbiome composition, but there was also an effect of origin site and an interaction between origin and destination site (Fig. 4A, PERMANOVA statistics in Table 3), but this was absent after two months (Fig. 4B, Table 3) and three months (Fig. 4C, Table 3). After two and three months, microbiomes still showed differences among all destination sites (Fig. 3). There was no difference in within-site variance among sites at any time point (betadisper ANOVA, *p*_1_ = 0.0912, *p*_2_ = 0.6264, *p*_3_ = 0.3975). Root bacterial communities from plants transplanted back to their home site differed in composition from undisturbed control plants at that site across all three months (PERMANOVA, Table 4, Fig. 3D–F). However, transplanted and control plants still overlap considerably in bacterial composition (Fig. 3D–F), and root communities from transplanted plants more closely resemble control root microbiomes than transplanted or control leaf microbiomes (Supplemental Fig. S9, PERMANOVA, *p*_rootvsleaf_ = 0.001).

**Table 3:** Results of PERMANOVA showing differences among transplanted root microbial communities (see Fig. 4).

		df	Sum Of Squares	R^2^	F-Statistic	Pr(> F)
After one month						
	Destination Site	3	12,417.762	0.259	18.196	**0.001**
	Origin Site	3	1235.735	0.026	1.811	**0.036**
	Destination : Origin	9	3086.977	0.064	1.508	**0.016**
	Residual	137	31,164.382	0.651	NA	NA
	Total	152	47,904.856	1	NA	NA
After two months						
	Destination Site	3	11,414.239	0.371	14.552	**0.001**
	Origin Site	3	600.808	0.020	0.766	0.704
	Destination : Origin	9	2295.097	0.075	0.975	0.49
	Residual	63	16,472.277	0.535	NA	NA
	Total	78	30,782.421	1	NA	NA
After three months						
	Destination Site	3	8991.433	0.360	8.760	**0.001**
	Origin Site	3	838.248	0.034	0.817	0.601
	Destination : Origin	8	2854.077	0.114	1.043	0.4
	Residual	36	12,317.357	0.493	NA	NA
	Total	50	25,001.115	1	NA	NA

Significant values are in bold.

**Table 4:** Results of PERMANOVA showing differences among based on variation in transplant status in roots (see Fig. 3D-F).

		**df**	**Sum Of Squares**	**R**^**2**^	**F-Statistic**	**Pr(> F)**
After one month						
	Site	3	7249.876	0.261	7.602	**0.001**
	Transplant Status	1	4336.789	0.156	13.643	**0.001**
	Site : Transplant Status	3	1605.829	0.058	1.684	0.056
	Residual	46	14,622.473	0.526	NA	NA
	Total	53	27,814.967	1	NA	NA
After two months						
	Site	3	4772.688	0.245	4.608	**0.001**
	Transplant Status	1	2723.638	0.140	7.888	**0.001**
	Site : Transplant Status	3	1596.102	0.082	1.541	0.081
	Residual	30	10,358.056	0.533	NA	NA
	Total	37	19,450.484	1	NA	NA
After three months						
	Site	3	3191.330	0.183	2.797	**0.003**
	Transplant Status	1	4062.769	0.233	10.682	**0.001**
	Site : Transplant Status	3	1788.470	0.103	1.567	0.08
	Residual	22	8367.592	0.481	NA	NA
	Total	29	17,410.161	1	NA	NA

Significant values are in bold.

We explored the interaction between destination and origin site on root microbiome after one month, by comparing microbiomes in all possible combinations of origin and destination site at this time point. Regardless of origin site, bacterial communities differed between all destination sites after one month (all pairwise results (*p*-values and R^2^) can be found in Supplemental Tables S5 and S6). However, origin site only influenced community composition for some origins at some destinations and only after one month. On roots of plants transplanted to MM, bacterial communities varied when comparing plants from MM vs. any other site while communities on roots of other sites did not differ from each other. The same pattern occurred at WP, where all plants transplanted from WP were distinct from all other sites and all other sites could not be distinguished from each other. At CC, bacterial communities roughly formed two groups with root bacterial communities—plants from CC and DB hosted similar communities and plants from MM and WP hosted a different distinct community. At DB, we saw no effect of origin site (all pairwise results *p*-values and R^2^s can be found in Supplemental Tables S7 and S8). There were no differences in alpha diversity among roots by destination after one or three months (negative binomial GLM, *p*_1_, *p*_3_ > 0.05), but after two months plants planted at WP had more ASVs (Fig. 4D–F; negative binomial GLM, *p*_destination_ = 0.007, *p*_origin_ = 0.023, *p*_destination:origin_ = 0.447; significant pairwise differences: *p*_destination CCvsWP_ = 0.011, *p*_destination MMvs WP_, *p*_origin MMvsWP_ = 0.023). Similarly, there were no differences in ASV richness among control and transplanted roots (ANOVA negative binomial GLM, *p*_1_ = 0.071, *p*_2_ = 0.572, *p*_3_ = 0.984) (Supplemental Fig. S8D–F), and variance within transplanted plants was typically the same as for the untransplanted control plants, except at three months (ANOVA, *p* = 0.164, *p*_2_ = 0.108, *p*_3_ = 0.0009).

### Most sites show phylogenetic clustering, degree of clustering varies by destination site

Given that all sites are within a few kilometers, and the high degree of tidal exchange within Bodega Harbor, all sites are likely exposed to a very similar pool of colonizing microbes; therefore we expect that the compositional differences among sites we observed (Fig. 2) are driven by local factors rather than dispersal limitation. We assessed whether, on average, community members were more or less related to each other than expected by chance by calculating the Nearest Relative Index (NRI), the average phylogenetic distance among all pairs of taxa, for each sample and comparing it to a random draw from the ASV pool. We provide details on how we performed these analyses in the Methods.

**Figure 5:**
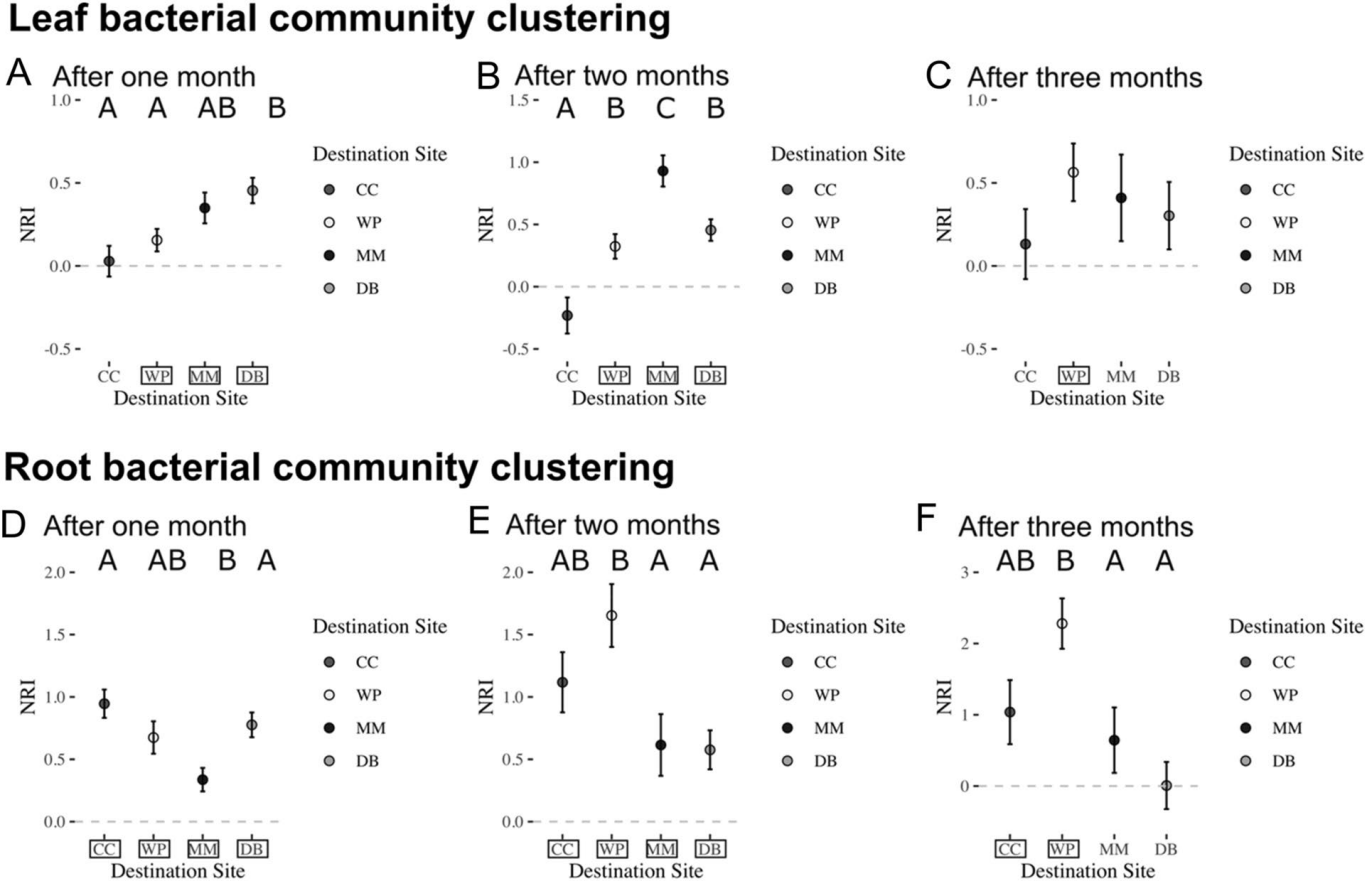
Leaf microbial communities were often phylogenetically clustered as measured by Net Relatedness Index (NRI) (mean + /- SE) by destination site after one (A), two (B), and three (C) months. Destination sites are indicated by abbreviation and colors indicated on the figure. A positive NRI indicates that communities at a site are phylogenetically clustered, a negative NRI indicates that a community is phylogenetically overdispersed. If a community’s NRI is significantly different from zero, we placed a box around the site name (see Supplemental Table S9 for more detailed statistics). Root microbial communities were consistently phylogenetically clustered as measured by Net Relatedness Index (NRI) (mean + /- SE) is shown by destination site after one (D), two (E), and three (F) months.

We found that leaf and root microbiomes were largely phylogenetically clustered; no community showed evidence of phylogenetic overdispersion in NRI (Fig. 5, Supplemental Table S9). Leaf microbiomes varied in the degree of clustering by site, with communities at the most oceanic sites being least clustered and those at sites that were warmer or with less water flow were most clustered (Fig. 5A–C). After one month, leaf bacterial communities were more clustered at DB than CC or WP; DB is the site furthest from the mouth of the harbor (Fig. 5A). After two months, we observed that communities at MM were the most clustered followed by DB and WP followed by CC which was neither clustered nor overdispersed; MM is the site with the least flow and CC is the site closest to the mouth of the harbor and the coolest site (Fig. 5B). There was no evidence of differences in phylogenetic clustering at any site after three months (Fig. 5C). These changes in clustering across months suggests temporal variation in ecological filtering, perhaps due to low tides being less extreme and more nocturnal during September than earlier in the summer. This could have reduced environmental differences due to exposure differences among sites.

Root bacterial communities were always clustered relative to null expectations and also showed differences in degree of phylogenetic clustering among sites via NRI (Fig. 5D–F) that were more consistent over time than we observed for leaves. Bacterial communities on roots at MM were less clustered than CC or DB sites after one month, after two and three months WP was more clustered than DB or MM. These differences may be associated with consistent water temperature differences across sites (DB and MM are warmer than CC and WP) or by sediment grain size (MM has a finer grain size than other sites).

### Individual phylogenetic balances reveal that the clades driving differences vary among sites; more in roots than leaves

To determine which ASVs drove the differences among destination sites and identify where these differences might occur, we employed a phylogenetic balance approach to identify the clades that varied among samples (see Methods for details). We found that differences among samples occurred throughout the tree: from the tips of trees containing only 2 ASVs (~ 0.02% of the ASVs in the pool), to near the base of the tree containing over 96% of ASVs. However, the median balance across samples was relatively small containing 3–4 ASVs within a node for leaf samples (depending on time point) and 5 ASVs per node in root samples (across time points). When we examined placement of nodes that defined differential balances, the phylogenetic placement of nodes was not different from a null distribution except for root bacterial communities after two and three months where nodes that differed were less basal (closer to tips) than expected (Supplemental Table S10). Overall, we found between 5 and 35 balances that identified each site from others within a time point. See Supplemental Figs. S2–S7 for specific balances that distinguished specific sites from others within a plant compartment and time point, and Supplemental Fig. S1 for interpretive guidance.

**Figure 6:**
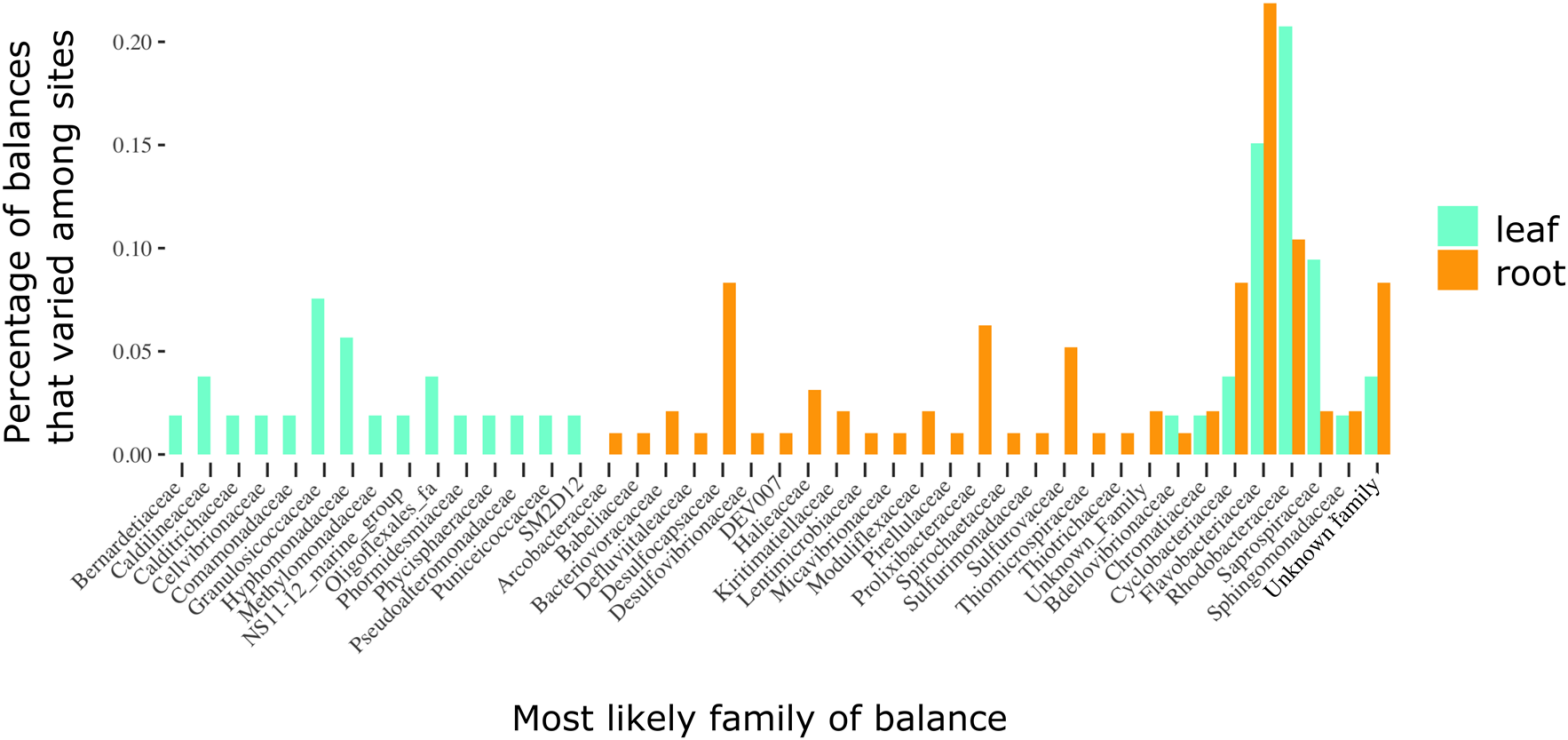
Microbial families contributing to differences among sites for both eelgrass leaves (teal) and roots (orange) based on identity of significant node (see Supplemental Information on balances and Supplemental Figs. S2–S7 for more details on these balance identities). We then compared families that varied in leaves among sites to those among roots below. A few families that varied among sites in both compartments, but most families that varied among sites were specific to leaves or roots (Fisher’s Exact Test p < 0.001).

As most of these nodes were at the level of differentiating among or within families (> 97% did not contain a node at the level of family or above), we examined which differentiating families were in each plant compartment. We found the families that differed in leaves were different than those that varied in roots (Fisher’s Exact Test *p* < 0.001, Fig. 6). The families most representative of differences in root microbiomes among sites include those putatively involved in sulfate reduction (including *Desulfocapsaceae*), sulfur oxidation (*Sulfurvaceae*) and nitrogen cycling (*Prolixbacteraceae*); but there were no consistent patterns of certain ASVs distinguishing certain sites across timepoints. In leaves, we did not identify families indicating processes that might differ among sites as we did in roots for sulfur and nitrogen cycling.

## Discussion

We found that geographic variation in eelgrass leaf and root microbiomes at the scale of a few kilometers and that plants transplanted among sites rapidly assumed the microbiome of the destination site, usually within a month. Root and leaf microbiomes were phylogenetically clustered within a site, but the degree of clustering varied, with sites more stressful for eelgrass (warmer, lower water flow, farther from the open ocean) showing a greater degree of clustering in leaf communities. Our balance analysis allowed us to further identify that many of the differences in communities among sites were at shallow nodes indicating fine taxonomic scale differences in communities. In conjunction with the shifts to match new environments and assuming that traits relevant to environmental tolerances are conserved, this suggests that microbiomes shift among phylogenetically similar taxa that differ in environmental tolerances but may have similar major metabolic capabilities that lead to similar functions within communities. Thus, we suggest that direct effects of environmental differences among sites drive these differences in microbial community assembly, though we cannot, as yet, conclusively identify the specific factors responsible. Furthermore, we highlight the differences in microbial community composition that can occur on small scales among seagrass beds within the same 5 km^2^ embayment.

Terrestrial leaf phyllosphere microbial communities are more phylogenetically clustered on faster growing trees, potentially due to stronger ecological filters or decreased time for microbial succession to play out^23^. With this in mind, our evidence of clustering in leaves is not surprising in eelgrass given the very quick turnover of leaf tissue (new leaves produced roughly every 14 days^40^) which would reduce the amount of time that competition would have to play out on leaf surfaces. In terrestrial phyllospheres^41^ competition is a major driver of community assembly given the low nutrient environments and likely competition for shared resources despite low nutrient environments often producing trait clustering^42^. While seagrass leaves are likely more nutrient rich environments than terrestrial plants due to the abundance of epiphytic algae growing on surfaces that exude DOC, the rapid leaf turnover likely reduces the influence of competitive interactions. We suggest that the relatively ephemeral nature of eelgrass leaves may facilitate adaptation to seasonal and temporal variation in environmental conditions (including interactions with other guilds living on seagrass blades). This also could partially explain lower levels of clustering we observed at CC—more random assembly could be driven by increased exposure time due to slower plant growth at this site (Kardish and Stachowicz unpublished data). This reduced plant growth rate may be related to cooler temperatures at CC compared to other sites (in July and August DB and MM were more clustered than CC and WP and also both have warmer water temperatures). In contrast, we found that clustering in the root microbiome was reduced at the warmer sites, (DB and MM) compared to cooler sites (WP and CC). We are unsure of the growth and turnover rate in roots which limits our ability to assess whether duration of environmental exposure plays a similar role for roots. However, we note that warming temperatures increase above ground growth at the expense of below ground growth in eelgrass^43^, which could lead to greater age of roots in warmer environments. Thus, in general rapid tissue turnover can provide one mechanism for minimizing priority effects in adjusting the host microbiome to a novel environment.

Additionally, there seem to be seasonal differences in leaf and root bacterial community assembly mechanisms. Leaf and root microbiome studies in other systems also show some evidence of predictable seasonal changes^8^, deterministic processes associated with tissue ontogeny^44^, and differences based on diversity of plant host species^45^. Our samples were taken at a standard position along the blade. Leaves grew more slowly in September compared to July and August (Kardish and Stachowicz unpublished data), thus, the leaf tissue we sampled would have been exposed for a longer time later in the season. This could provide more time for biotic interactions to play out, decreasing the relative importance of environmental filtering leading to more random rather than clustered assembly in September. However, this could also be driven by shallower, shorter, more nocturnal tides in September decreasing variability within environments among these intertidal beds, speeding succession in these communities. Further work would be needed to disentangle which seasonal effects are changing community assembly mechanisms. Seasonal differences are critical to understanding differences in community assembled differences across seasons are understudied in community phylogenetics even in non-microbial systems (see^46^ for an example in tropical fish communities) and should be particularly prominent in systems like this where changing plant environment changes the microbiome in less than a month–a similar time scale to that of seasonal environmental change.

One caveat to these conclusions for root microbiome changes is that we did find differences in root microbiomes between back-transplanted and untransplanted controls, though no such effects were found for leaf microbiomes. The root specific effects could be caused by our methods of transplantation (which involved attachment to plastic mesh to track shoots and a several day period in seawater (with roots out of sediment)) being more stressful to roots and their bacterial communities than leaves or their communities resulting in selection for different microbial communities upon transplantation. Alternatively, more rapid turnover in leaf than root tissue could mean that transplant effects in leaves, if they occurred lasted less than a few weeks. Relatively few previous reciprocal transplant studies of host microbiomes have included transplant controls (but see^47^ for an example with transplant controls in DNA fingerprinting identifying differences between transplants and undisturbed controls in fucoid seaweeds), and our work highlights that this should be an important part of experimental designs. Transplanting individual plants is an important mechanism of restoration in eelgrass^48,49^, so understanding transplant effects on the microbiome is of practical interest if changes result in functional differences. That said, root communities on transplanted plants still strongly resemble control root microbial communities (Fig. S9) and reproduce the broad destination site effects found in the transplanted plants. Thus, we think that our conclusion that root microbiomes vary among sites and root assemblages shift with local environmental conditions remain robust.

Ultimately, understanding the consequences of these changes in leaf and root microbiomes for eelgrass fitness will require functional genomic studies or experimental additions, which are beyond the scope of this paper. At this point, we are limited to inferring potential changes in function associated with which phylogenetic balances are most responsible for differences in community composition in space or time. While we identified many balances that we used to identify different sites within time points, they varied by time point and site (i.e., we did not consistently identify the same balances differentiating certain sites). We found that the nodes that distinguished sites were not differently placed on the tree than expected by chance, so the changes we observed occurred at all taxonomic levels. When we examined the families that distinguished leaf and root bacterial communities we found several differences, but perhaps most notably were differences within the family *Desulfocapsaceae* in roots. *Desulfocapsaceae* is a family containing known sulfate reducers associated with the top layers of marine sediments was found almost exclusively as an identifying balance in root bacterial community samples^50^. This bacterial family distinguished roots at CC, MM, and DB from other sites after one month and multiple balances distinguished DB from other sites at time points two and three. This potentially indicates that sulfur cycling at DB requires different members of *Desulfocapsaceae* that are perhaps adapted to the warmer temperatures at this site. This identification of a family with conserved traits (e.g., sulfur metabolism^51^) that is known to have a diversity of other tolerances (e.g., temperature^52,53^) does also suggest that this kind of phylogenetic approach is useful here in identifying groups with some conserved and some variable phylogenetic traits that could contribute to differences in environmental matching^51,54^. These traits of members of *Desulfocapsaceae* could potentially be explored through further metagenomic work exploring different roles and abilities of members of *Desulfocapsaceae* at these different sites or through culture-based approaches. Our previous work has identified many bacteria related to sulfur cycling enriched on seagrass roots, and especially noted the presence of sulfur oxidizers enhanced compared to sediments^31^. More direct experimental work with different members of this family would be necessary to tease apart any different functions or differences in sulfur reducing abilities, they might have to determine if these are functionally redundant or if they result in differences to functional potential as well.

Changes in microbiomes potentially buffer hosts against stressful environments^55,56^, but understanding the pace of these shifts is critical for assessing whether the host can survive the period of mismatch. We found that eelgrass microbiomes rapidly shift (< 1 month) in response to novel environments, and that these microbiomes, especially in roots, are distinguishable among sites based on taxa associated with key functions of nitrogen and sulfur metabolism. This suggests the potential for the microbiome to buffer the host against a changing environment through rapid community re-assembly. Using manipulative experiments to assess microbiome functionality in field settings remains a challenging and important goal, and metagenomics studies, while powerful, can be limited in sample size due to costs. In this context, our approach of host transplantation combined with community phylogenetic and phylogenetic balance approaches has suggested some testable functional hypotheses and provides a viable approach for making progress on the functionality of microbiomes in non-model systems in field situations.

## Methods

### Reciprocal Transplant and Field Methods

We collected individual eelgrass shoots and reciprocally transplanted them among four seagrass beds within Bodega Harbor (California) at Campbell Cove (“CC”, 38˚18′36″ N, 123˚3′33″ W), Mason’s Marina (“MM”, 38˚20′10″ N, 123˚3′31″ W), Westside Park (“WP”, 38˚19′7″ N, 123˚3′12″ W), and Doran Beach (“DB”, 38˚19′21″ N, 123˚2′38″ W). The sites represent discrete seagrass beds, but range in distance from each other from 0.9 to 3.0 km. Plants at these sites have different phenotypic characteristics (e.g., nutrient uptake, morphological traits, growth traits, photosynthetic traits, and phenolics) when grown in common gardens^39^ and show genetic differentiation at both neutral loci^38,39^ and coding loci associated with temperature tolerance (Scheibelhut, Grosberg, Stachowicz, and Bay unpublished data). MM is the most distinct genetically and environmentally, potentially driven by finer sediment with higher organics as well as reduced water flow. CC is closest to the harbor mouth with high flow and sandy sediment and (along with WP) has the coolest water temperature of these sites. DB is our warmest site about 2˚C warmer in mean temperature than CC and WP and is also the site least impacted by human presence (all other sites are routinely visited by recreation clam fishers. In the summer of 2015, the mean temperature of these sites ranged from 15.9 to 18.1 ˚C (instantaneous temperature across sites temperature ranged from 12.4 to 21.8 ˚C by HOBO loggers) (See Supplemental Table S11 for temperature data by site).

We took collected plants to Bodega Marine lab, standardized shoot length to 30 cm and rhizome length to 5 cm, and attached plants to vexar mesh blind to site of origin. Then we planted twelve vexar mesh screens with 16 plants at each of the four sites within an existing eelgrass bed with 1 m in between screens (plants growing immediately below screens were removed). Our design was fully reciprocal—four plants from each site were attached to each mesh screen. Using these screens allowed us to track individual plants planted within existing eelgrass beds, and remove them at the end of our experiment. Two weeks before sampling for microbial samples, we marked plants to standardize the age of microbial samples using the hole punch method^57,58^.

We sampled plants destructively every month for three months during a pre-dawn low tide. When sampling, we randomly selected 3 screens at each site and measured and sampled from every (remaining, non-flowering) plant on those screens. We rinsed plants in seawater on site before sampling to remove loose epiphytes and sediment. We collected approximately 10 roots from each plant along with a 2–3 cm section of the oldest non-senescent leaf immediately distal to the marks made within the leaf sheaf two weeks prior. Because the meristem is within the sheath, this means that all leaf tissue sampled being approximately the same age and exposed outside the leaf sheath for approximately 2 weeks. This also means that the tissue sampled had not been exposed to the external environment from the origin site at the time of transplant but was growing adjacent to leaf tissue that was growing with an intact origin-site microbiome. We immediately froze all microbe samples on dry ice and stored at -80˚C. Additionally, from each plot we took parallel microbial samples from four control plants (leaves and roots) of similar size that had not been transplanted from around the perimeter of the experimental plots to serve as controls for the effect of the transplantation process on the microbiome.

### Molecular methods

We extracted DNA with the MoBio PowerSoil DNA kit from the surface of the leaves and roots and from scaled samples of samples. To get microbes from only the plant surface, we vortexed each frozen sample with 500ul of MilliQ water and then added that liquid to the bead tubes and proceeded with the standard extraction protocol (full protocol available at github.com/mkardish/Transplants/Lab_Protocols). We lost DNA extractions for leaf samples transplanted to CC from MM and WP due to laboratory error so these are missing from the analysis. Due to loss of some plants at later time points, we sampled fewer plants at later vs. early time points. Final replicate count (per timepoint, sample type, origin site, destination site), can be found in Supplemental Table S12. We amplified and sequenced the V4–V5 region of the 16S rRNA gene on an Illumina MiSeq on an Illumina MiSeq to identify bacteria present with primers 515F and 926R^59,60^ at the Integrated Microbiome Resource at Dalhousie University.

### Bioinformatic analysis

We ran all bioinformatic and statistical analyses in R (version 3.6.1). We used a standard dada2 pipeline to error check our reads and to identify amplicon sequence variants^61^. We used only forward reads in our subsequent analyses (280 base pairs). We identified ASV taxonomy based on the SILVA database^62^ and built a phylogeny of ASVs using alignments built with DECIPHER^63^ then a tree built with FastTree2^64^ then converted to ultrametric^65^. We then rooted the bacterial tree with an archaeal outgroup^66,67^.

### Statistical analysis

We determined differences among groups of samples based on Euclidean distances after phylogenetic isometric log-transform implemented in the R-package “philr” order to analyze the compositional changes in our dataset based on phylogenetic similarity^68^. This analysis tests the differences in weighting of various regions of a phylogenetic tree rather than considering each ASV independently. We used PERMANOVA to determine differences among sample types (among sample types, sample timepoint, sample origin site and destination site) on ASVs present in at least 2 samples. We tested homogeneity of group dispersions with the betadispr function in ‘vegan’, we then assessed significance with an ANOVA test followed by Tukey’s Post-Hoc Test. To measure bacterial richness, we rarified all samples to 1980 reads, calculated the number of “Observed ASVs”, repeated this 200 times, and then used the average as our measure of bacterial richness in a sample^69^. We used pairwise-posthoc analyses to assess support for differences in observed ASV richness among groups.

We examined compositional differences among sites and treatments, by assessing phylogenetic clustering and overdispersion of groups relative to random draws from regional and local pools. We assume that some bacterial traits, especially those associated with major metabolic processes, are phylogenetically conserved^25,26^, and thus conclude that phylogenetic clustering is associated with environmental filtering and dispersion with competitive forces, resource partitioning, or cross-feeding. We also examined the individual balances driving the differences in the communities we observed by identifying balances that made communities distinct from others.

We present data on the net relatedness index (NRI) of a community which is the negative of the standardized effect size (SES) of the calculated mean pairwise distance (MPD) compared to a null distribution. The NRI thus represents the average phylogenetic distance between all members of a community relative to an expected null value^70^ such that positive values represent phylogenetic clustering and negative values represent overdispersion relative to expectations. We did this through the R package MicEco which implements a parallelized version of picante^71,72^. We used an independent swap null model in order to maintain sample richness and species occurrence frequency and compared MPD from our samples to 999 random draws. We only show data from abundance weighted models, but non-abundance weighted models show the same patterns. We tested for differences SES among sites using ANOVAs or Kruskal Wallis tests when the residuals were not normal.

In addition to the clustering analysis, we also assessed which phylogenetic balances differed between groups of interest. This analysis uses multinomial logistic regression implemented in glmnet in order to identify which branches of the phylogeny differ in their representation among groups (Supplemental Fig. S1)^68,73^. To select significant balances, we repeated cross validation procedures selecting the minimum lambda 100 times for each comparison to establish balances that best represented sites. We report how many iterations we found any balance shown across these trials. Finally, we summarized these balances, examining which families differed in leaves and roots across all time points. We also used a t-test to examine whether the position of distinguishing nodes on the phylogenetic tree were different than we would have expected due to chance alone (e.g., were shallow or deep branches more likely to contribute to compositional differences among treatments).

## Supplementary Information

Supplementary Information.

## Data Availability

All data and code can be found at https://github.com/mkardish/Transplants and sequences have been deposited under the NCBI BioProject ID PRJNA731931.
