# Supplementary material for: Local environment drives rapid shifts in composition and phylogenetic clustering of seagrass microbiomes
Source: Sci Rep. 2023 Mar 4;13:3673. doi: 10.1038/s41598-023-30194-x (PMC9985655; doi:10.1038/s41598-023-30194-x)

Supplemental Table S1: P-values from pairwise PERMANOVA of leaf samples at T1 testing the effects of destination site from particular origin sites. All values are fdr-corrected and bold values are significant at 0.05 after correction.

| Destination Sites Compared | from CC | from DB | from MM | from WP |
| --- | --- | --- | --- | --- |
| CC and DB | **0.0018** | **0.0018** | NA | NA |
| CC and MM | **0.0018** | **0.0018** | NA | NA |
| CC and WP | **0.0018** | **0.0018** | NA | NA |
| DB and MM | **0.0018** | **0.0018** | **0.0018** | **0.0245** |
| DB and WP | **0.0018** | **0.0018** | **0.0018** | **0.0018** |
| MM and WP | **0.0018** | **0.0018** | **0.026** | **0.0267** |

Supplemental Table S2: R^2^ from pairwise PERMANOVA of leaf samples at T1 testing the effects of destination site from particular origin sites. Bold values were significant after fdr-correction (see Supplemental Table S1 for p-values)

| Destination Sites Compared | from CC | from DB | from MM | from WP |
| --- | --- | --- | --- | --- |
| CC and DB | **0.607** | **0.452** | NA | NA |
| CC and MM | **0.521** | **0.406** | NA | NA |
| CC and WP | **0.339** | **0.215** | NA | NA |
| DB and MM | **0.214** | **0.231** | **0.278** | **0.123** |
| DB and WP | **0.484** | **0.334** | **0.201** | **0.348** |
| MM and WP | **0.416** | **0.31** | **0.174** | **0.139** |

Supplemental Table S3: P-values from pairwise PERMANOVA of leaf samples at T1 testing the effects of origin site at particular destination sites. All values are fdr-corrected and bold values are significant at 0.05 after correction.

| Origin Site Compared | to CC | to DB | to MM | to WP |
| --- | --- | --- | --- | --- |
| CC and DB | 0.0944 | 0.1356 | 0.4216 | 0.3628 |
| CC and MM | NA | **0.0035** | **0.026** | 0.0519 |
| CC and WP | NA | 0.2027 | 0.0829 | 0.2493 |
| DB and MM | NA | **0.0068** | 0.0546 | 0.0931 |
| DB and WP | NA | 0.856 | 0.0735 | 0.1003 |
| MM and WP | NA | 0.22 | 0.0787 | 0.0768 |

Supplemental Table S4: R^2^ from pairwise PERMANOVA of leaf samples at T1 testing the effects of origin site at particular destination sites. Bold values were significant after fdr-correction (see Supplemental Table S3 for p-values)

| Origin Sites Compared | to CC | to DB | to MM | to WP |
| --- | --- | --- | --- | --- |
| CC and DB | 0.083 | 0.067 | 0.051 | 0.06 |
| CC and MM | NA | **0.131** | **0.097** | 0.163 |
| CC and WP | NA | 0.07 | 0.102 | 0.066 |
| DB and MM | NA | **0.098** | 0.137 | 0.122 |
| DB and WP | NA | 0.038 | 0.129 | 0.078 |
| MM and WP | NA | 0.067 | 0.107 | 0.139 |

Supplemental Table S5: P-values from pairwise PERMANOVA of root samples at T1 testing the effects of destination site at particular origin sites. All values are fdr-corrected and bold values are significant at 0.05 after correction.

| Destination Sites Compared | from CC | from DB | from MM | from WP |
| --- | --- | --- | --- | --- |
| CC and DB | **0.0027** | **0.0027** | **0.0094** | **0.006** |
| CC and MM | **0.0027** | **0.0027** | **0.0027** | **0.0027** |
| CC and WP | **0.006** | **0.006** | **0.0048** | **0.0162** |
| DB and MM | **0.0027** | **0.0027** | **0.0048** | **0.0103** |
| DB and WP | **0.0048** | **0.0027** | **0.0103** | **0.0027** |
| MM and WP | **0.0027** | **0.0103** | **0.0027** | **0.0027** |

Supplemental Table S6: R^2^ from pairwise PERMANOVA of root samples at T1 testing the effects of destination site at particular origin sites. Bold values were significant after fdr-correction (see Supplemental Table S5 for p-values).

| Destination Sites Compared | from CC | from DB | from MM | from WP |
| --- | --- | --- | --- | --- |
| CC and DB | **0.287** | **0.208** | **0.175** | **0.254** |
| CC and MM | **0.42** | **0.295** | **0.43** | **0.376** |
| CC and WP | **0.187** | **0.172** | **0.135** | **0.137** |
| DB and MM | **0.352** | **0.26** | **0.291** | **0.218** |
| DB and WP | **0.213** | **0.195** | **0.188** | **0.282** |
| MM and WP | **0.213** | **0.173** | **0.418** | **0.375** |

Supplemental Table S7: P-values from pairwise PERMANOVA of root samples at T1 testing the effects of origin site at particular destination sites. All values are fdr-corrected and bold values are significant at 0.05 after correction.

| Origin Sites Compared | to CC | to DB | to MM | to WP |
| --- | --- | --- | --- | --- |
| CC and DB | 0.4189 | 0.3727 | 0.5369 | 0.456 |
| CC and MM | *0.0747* | 0.2437 | **0.0385** | 0.1404 |
| CC and WP | **0.0189** | 0.3826 | 0.2808 | **0.042** |
| DB and MM | **0.046** | 0.759 | **0.046** | 0.2175 |
| DB and WP | *0.0518* | 0.6071 | 0.3353 | **0.015** |
| MM and WP | 0.4757 | 0.5126 | **0.0077** | **0.046** |

* Note: Many of the p-values varied slightly between different permutations of data. We have highlighted sets here that were close to significance after correction as their variance allows us to best understand the community patterns.

Supplemental Table S8: R^2^ from pairwise PERMANOVA of root samples at T1 testing the effects of origin site at particular destination sites. Bold values were significant after fdr-correction (see Supplemental Table 7 for p-values).

| Origin Sites Compared | to CC | to DB | to MM | to WP |
| --- | --- | --- | --- | --- |
| CC and DB | 0.287 | 0.208 | 0.175 | 0.254 |
| CC and MM | *0.42* | 0.295 | **0.43** | 0.376 |
| CC and WP | **0.187** | 0.172 | 0.135 | **0.137** |
| DB and MM | **0.352** | 0.26 | **0.291** | 0.218 |
| DB and WP | *0.213* | 0.195 | 0.188 | **0.282** |
| MM and WP | 0.213 | 0.173 | **0.418** | **0.375** |

Supplemental Table S9: Net Relatedness Index (NRI) values differ from zero across most sites, time points, and sample types. Here a significant p-value indicates that a site has an NRI are significantly different from zero. In our case, all of these indicate clustered communities at a site (see Figures 4 and 5).

| Sample Type | Time | Site | p-value NRI |
| --- | --- | --- | --- |
| Leaf | 1 | CC | 0.762 |
|  |  | DB | **<0.001** |
|  |  | MM | **0.001** |
|  |  | WP | **0.028** |
|  | 2 | CC | 0.135 |
|  |  | DB | **<0.001** |
|  |  | MM | **<0.001** |
|  |  | WP | **0.003** |
|  | 3 | CC | 0.544 |
|  |  | DB | 0.174 |
|  |  | MM | 0.142 |
|  |  | WP | **0.006** |
| Root | 1 | CC | **<0.001** |
|  |  | DB | **<0.001** |
|  |  | MM | **0.001** |
|  |  | WP | **<0.001** |
|  | 2 | CC | **<0.001** |
|  |  | DB | **0.001** |
|  |  | MM | **0.024** |
|  |  | WP | **<0.001** |
|  | 3 | CC | **0.038**** |
|  |  | DB | 0.984 |
|  |  | MM | 0.186 |
|  |  | WP | **<0.001** |

** ns after fdr correction

Supplemental Table S10: Nodes that are significantly different among sites are not more or less phylogenetically shallow than expected by chance, except in roots at later time points. Red is the observed distribution of tip placement as shown in Figures S2-S7, black is the null distribution based on all nodes present in samples at those time points. Only roots at T2 and T3 show slightly less shallow than expected. All distances are log-normalized.

| Sample Type | Time |  | t-test |  |  |
| --- | --- | --- | --- | --- | --- |
| Leaf | 1 | 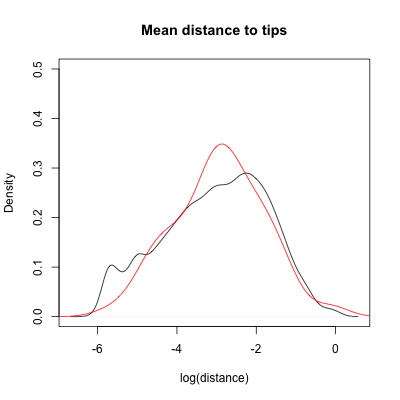 | t = - 0.81862,  df = 88.148,  p-value = 0.415 |  |  |
|  |  |  |  |  |  |
|  |  |  |  |  |  |
|  |  |  |  |  |  |
|  | 2 | **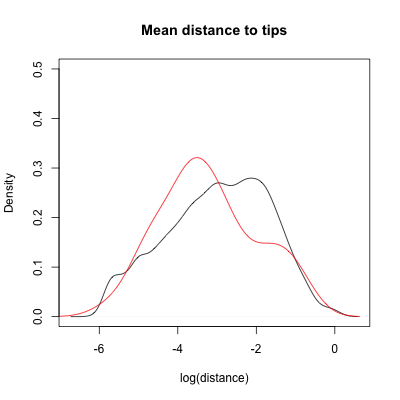** | t = 1.2126,  df = 39.488,  p-value = 0.2325 |  |  |
|  |  |  |  |  |  |
|  |  |  |  |  |  |
|  |  |  |  |  |  |
|  | 3 | **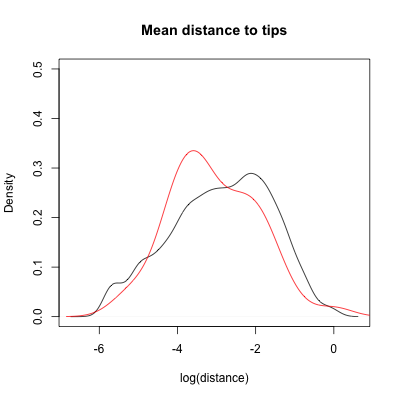** | t = 1.1535,  df = 46.008,  p-value = 0.2547 |  |  |
|  |  |  |  |  |  |
|  |  |  |  |  |  |
|  |  |  |  |  | |

| Sample Type | Time |  | t-test |  |
| --- | --- | --- | --- | --- |
| Root | 1 | **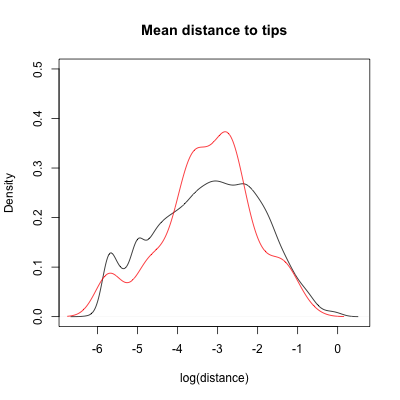** | t = 0.18297,  df = 68.81,  p-value = 0.8554 |  |
|  |  |  |  |  |
|  |  |  |  |  |
|  |  |  |  |  |
|  | 2 | **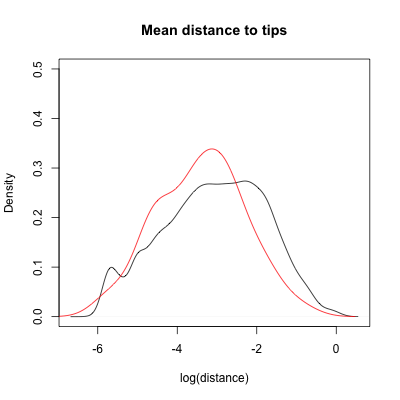** | **t = 2.2451,**  **df = 54.835,**  **p-value = 0.02881** |  |
|  |  |  |  |  |
|  |  |  |  |  |
|  |  |  |  |  |
|  | 3 | 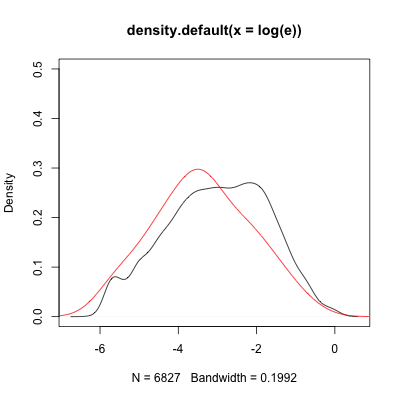 | **t = 2.3334,**  **df = 45.165,**  **p-value = 0.02414** |  |
|  |  |  |  |  |
|  |  |  |  |  |
|  |  |  |  |  |

Supplemental Table S11: Temperature and other notes about site characteristics.

|  | **Campbell Cove**  **(CC)** | **Westside**  **Park**  **(WP)** | **Mason’s Marina**  **(MM)** | **Doran**  **Beach**  **(DB)** |
| --- | --- | --- | --- | --- |
| Temperature summer 2015 (7/8 -9/23) ˚C  mean +/- SD  range  *Measured in this study* | 15.86 +/- 1.21  12.5 - 19.66 | 16.27 +/- 1.24  12.4 - 20.62 | N/A  (HOBO data collector lost) | 18.07 +/- 1.38  14.42 - 21.76 |
| Temperature summer 2019 (7/17 - 8/30) ˚C  mean +/- SD  range  *Stachowicz unpublished data* | 14.86 +/- 1.57  10.59 - 18.83 | 15.34 +/- 1.56  10.34 - 21.11 | 15.81 +/- 1.48  12.44 - 18.83 | N/A  (not measured in this study) |
| Other site notes | Closest to mouth of the harbor, high flow, sandy sediment | Site of many eelgrass experiments at Bodega Bay | Restored site, finer sediment grain size | Furthest from clamming activity |

Supplemental Table S12: The final counts of sequenced replicates in each sample type, timepoint, transplant site, destination site combination. This represents a total of 307 microbial samples from seagrass leaves and 330 root samples.

| Transplant Site | Destination Site | Final Sequenced Replication for leaves | | | Final Sequenced Replication for roots | | |
| --- | --- | --- | --- | --- | --- | --- | --- |
|  |  | One Month | Two Months | Three Months | One Month | Two Months | Three Months |
| CC | CC | 10 | 5 | 4 | 10 | 5 | 4 |
|  | DB | 9 | 5 | 3 | 9 | 5 | 4 |
|  | MM | 0* | 2 | 2 | 9 | 2 | 2 |
|  | WP | 0* | 2 | 4 | 9 | 2 | 4 |
| Unmoved controls from CC | | 4 | 4 | 4 | 4 | 4 | 4 |
| DB | CC | 10 | 6 | 0 | 10 | 6 | 0 |
|  | DB | 10 | 6 | 4 | 10 | 6 | 4 |
|  | MM | 10 | 6 | 4 | 10 | 6 | 4 |
|  | WP | 9 | 6 | 1 | 9 | 6 | 1 |
| Unmoved controls from DB | | 4 | 4 | 4 | 4 | 4 | 3 |
| MM | CC | 9 | 3 | 3 | 10 | 3 | 3 |
|  | DB | 9 | 6 | 4 | 10 | 6 | 4 |
|  | MM | 8 | 5 | 4 | 8 | 5 | 4 |
|  | WP | 10 | 3 | 2 | 10 | 3 | 2 |
| Unmoved controls from MM | | 4 | 4 | 4 | 4 | 4 | 4 |
| WP | CC | 9 | 6 | 4 | 9 | 6 | 4 |
|  | DB | 10 | 6 | 4 | 10 | 6 | 4 |
|  | MM | 7 | 6 | 4 | 10 | 6 | 4 |
|  | WP | 10 | 6 | 3 | 10 | 6 | 3 |
| Unmoved controls from WP | | 4 | 4 | 4 | 4 | 4 | 4 |

**Supplemental Figures**

Supplemental Figure S1. Interpreting phylogenetic balances. Each balance identified here is a single node in the tree of microbial communities where one site was differentially weighted compared to all other sites. Here for this node within the bacterial family Cyclobacteriaceae, CC has two ASVs in this family upweighted compared to the seven ASVs on the other side of the node. All other sites had higher relative abundance of the ASVs on the other side of the node. As we ran the glmnet model 100 times, we represent the percentage of times that this ASV appeared significant in this comparison (here 100%). The error bars represent the standard error of the predicted balance weight.

**
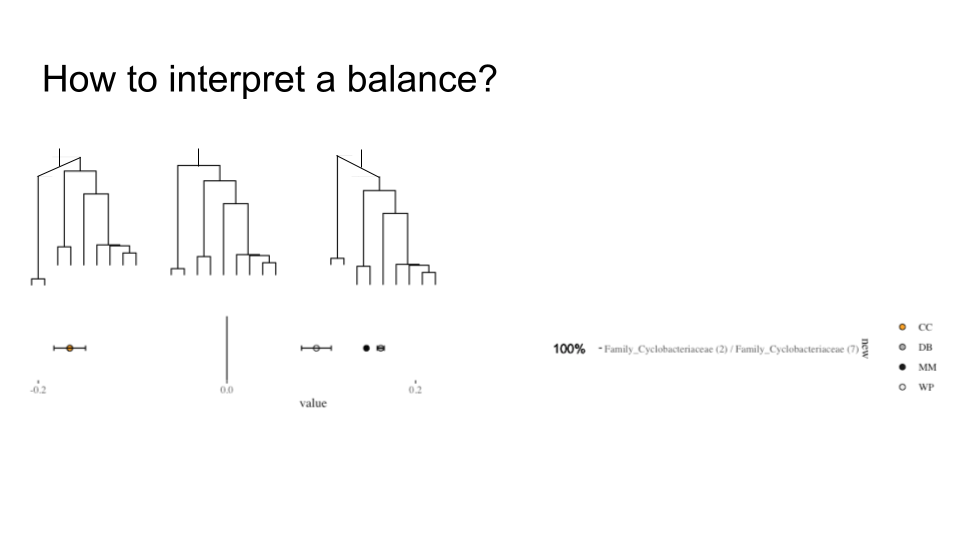

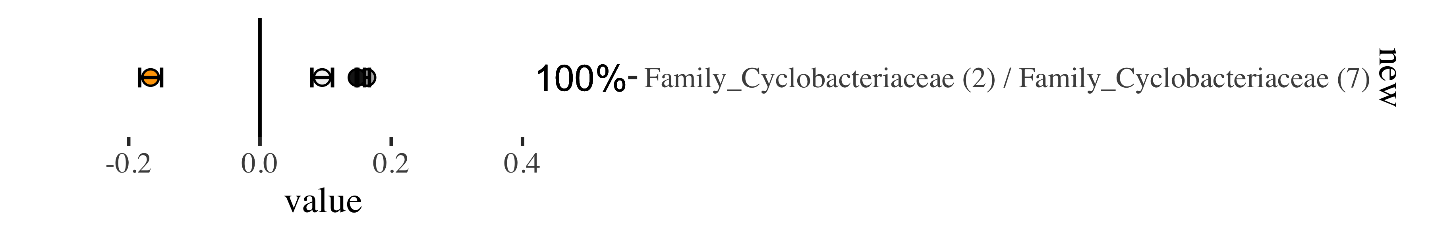
**

Supplemental Figure S2: After one month, we identified 14 balances distinguishing leaf bacterial communities at CC, 15 balances at DB, 35 at MM and 27 at WP. See Figure S1 for details on symbols used and interpretation of results.

**
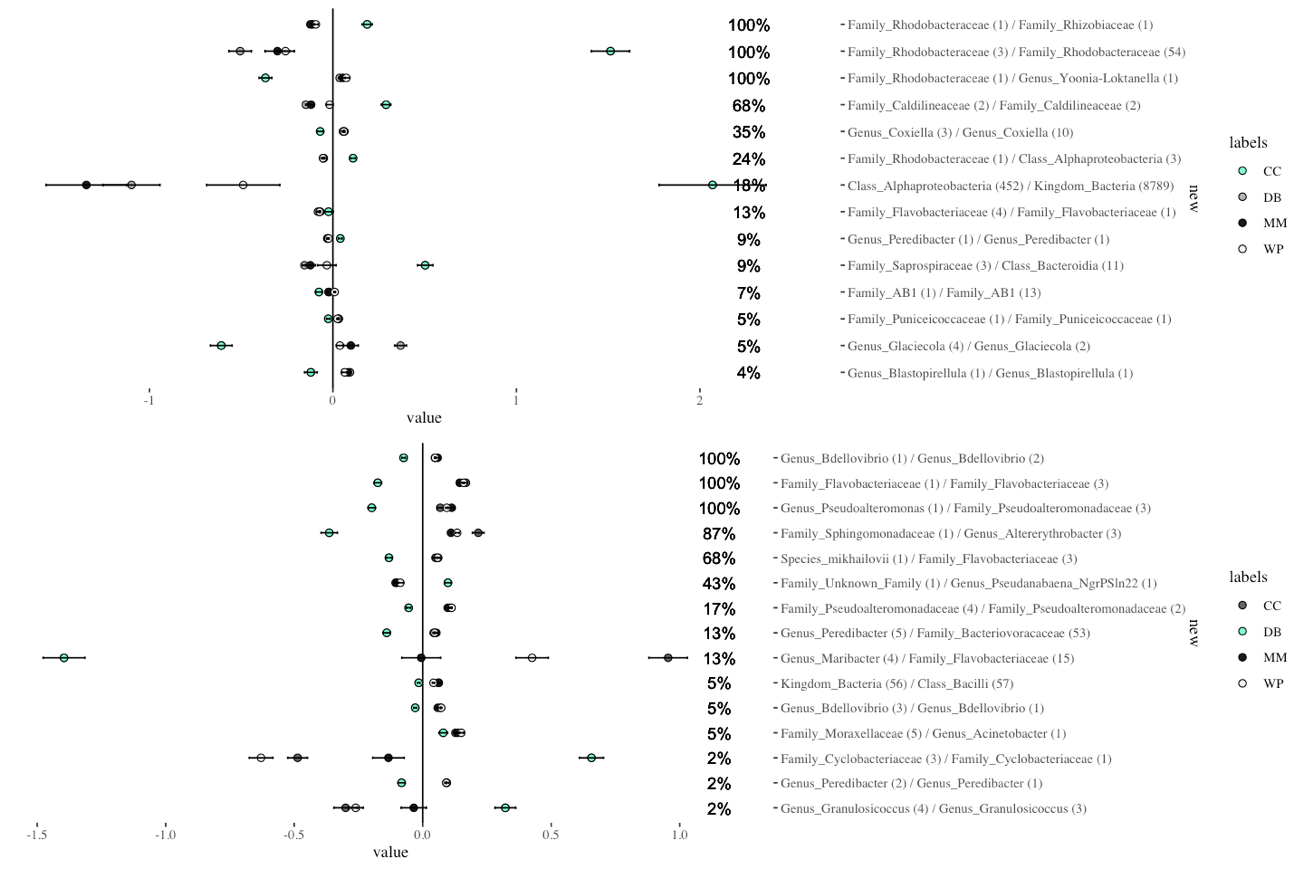

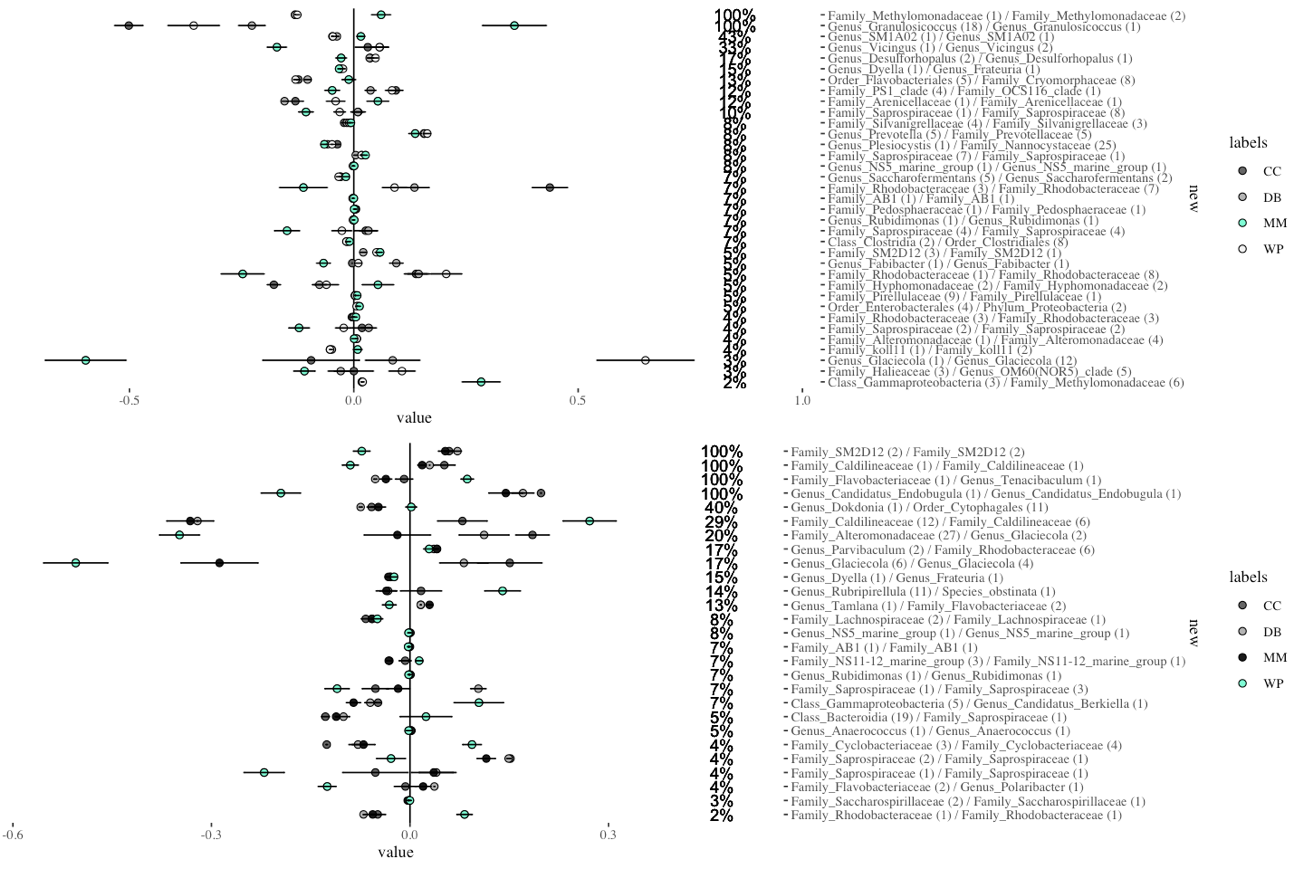
**

Supplemental Figure S3: After two months, we identified 9 balances distinguishing leaf bacterial communities at CC, 11 balances at DB, 11 at MM and 9 at WP. See Figure S1 for details on symbols used and interpretation of results.

**
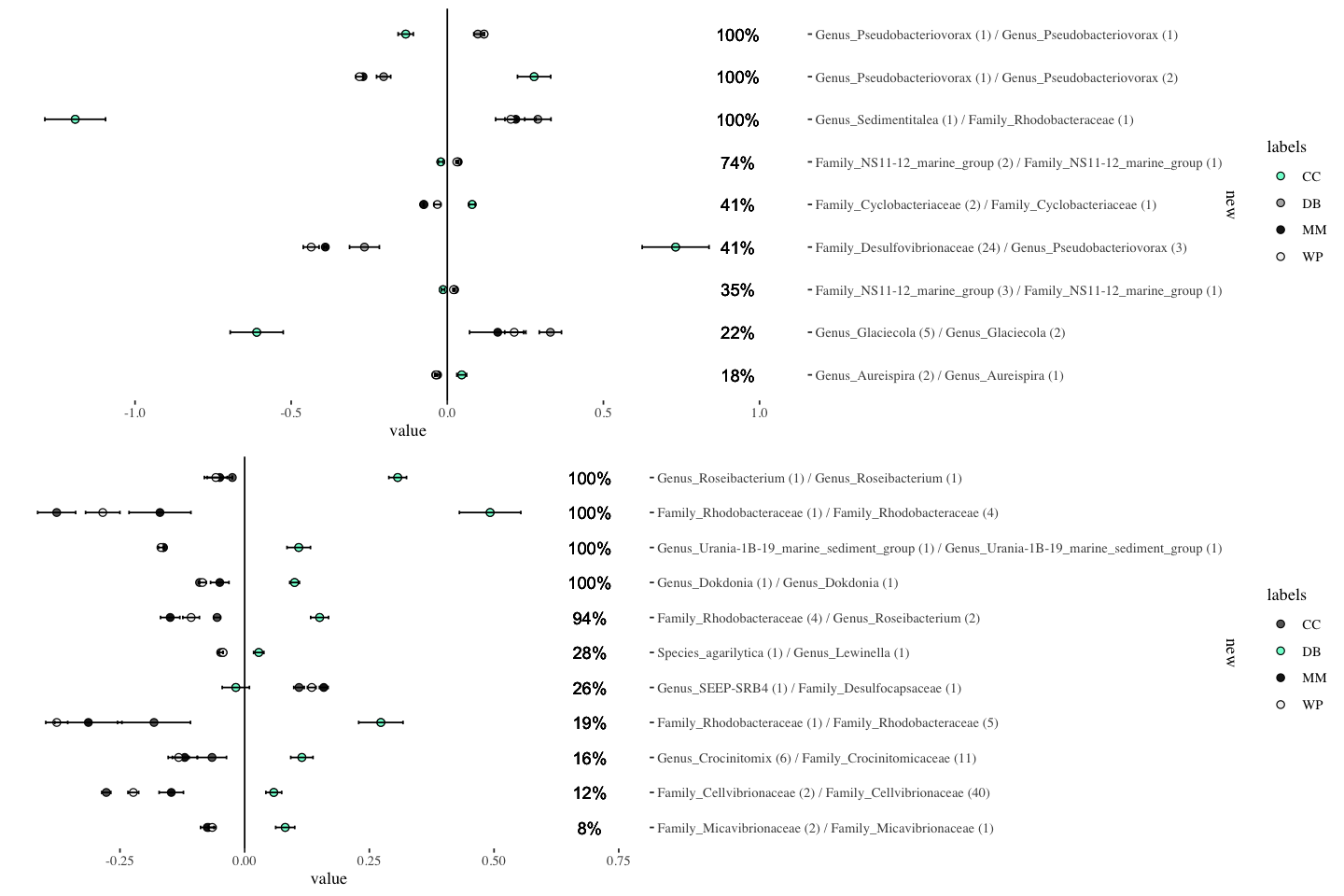

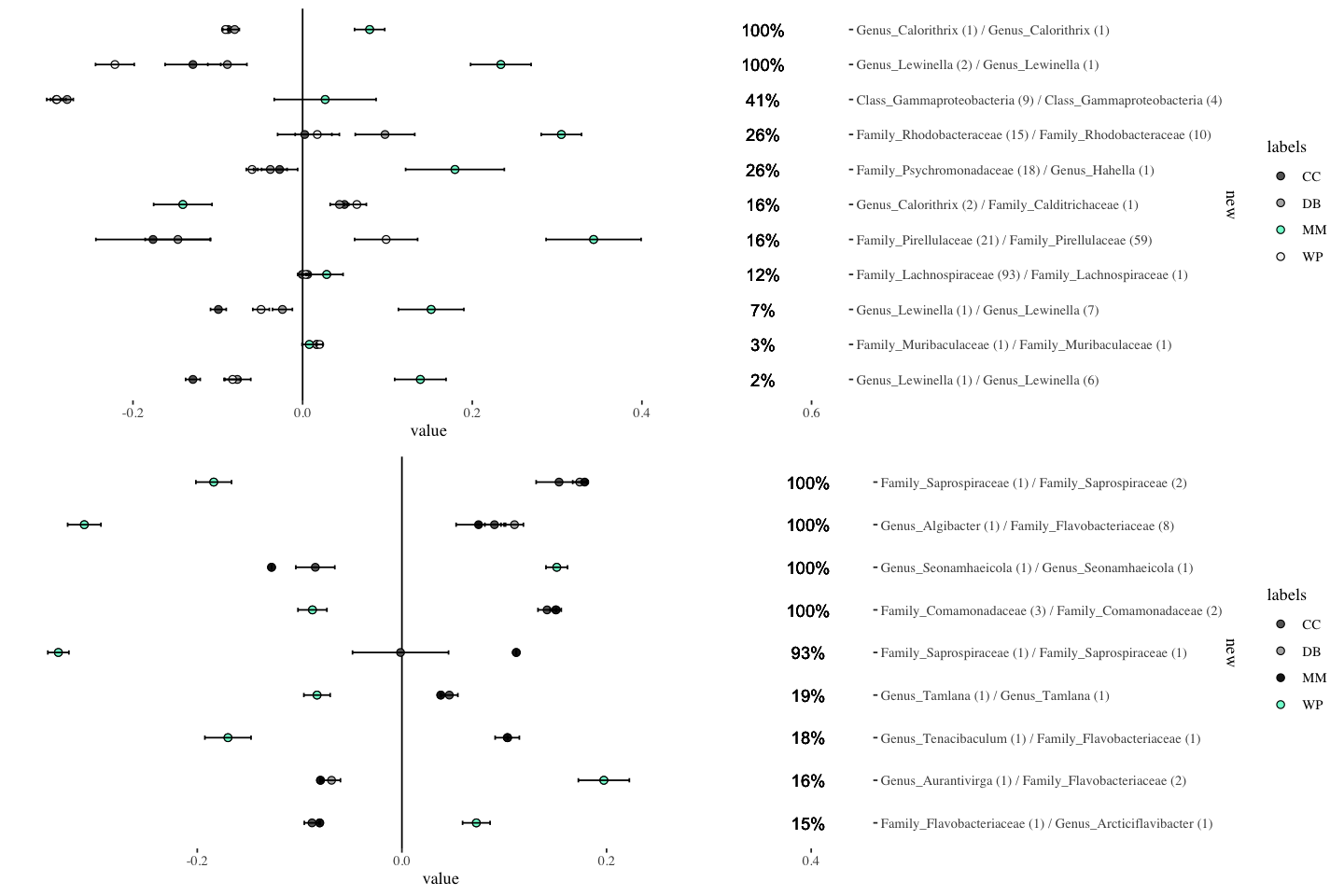
**Supplemental Figure S4: After three months, we identified 8 balances distinguishing leaf bacterial communities at CC, 11 balances at DB, 16 at MM and 11 at WP. See Figure S1 for details on symbols used and interpretation of results.

**
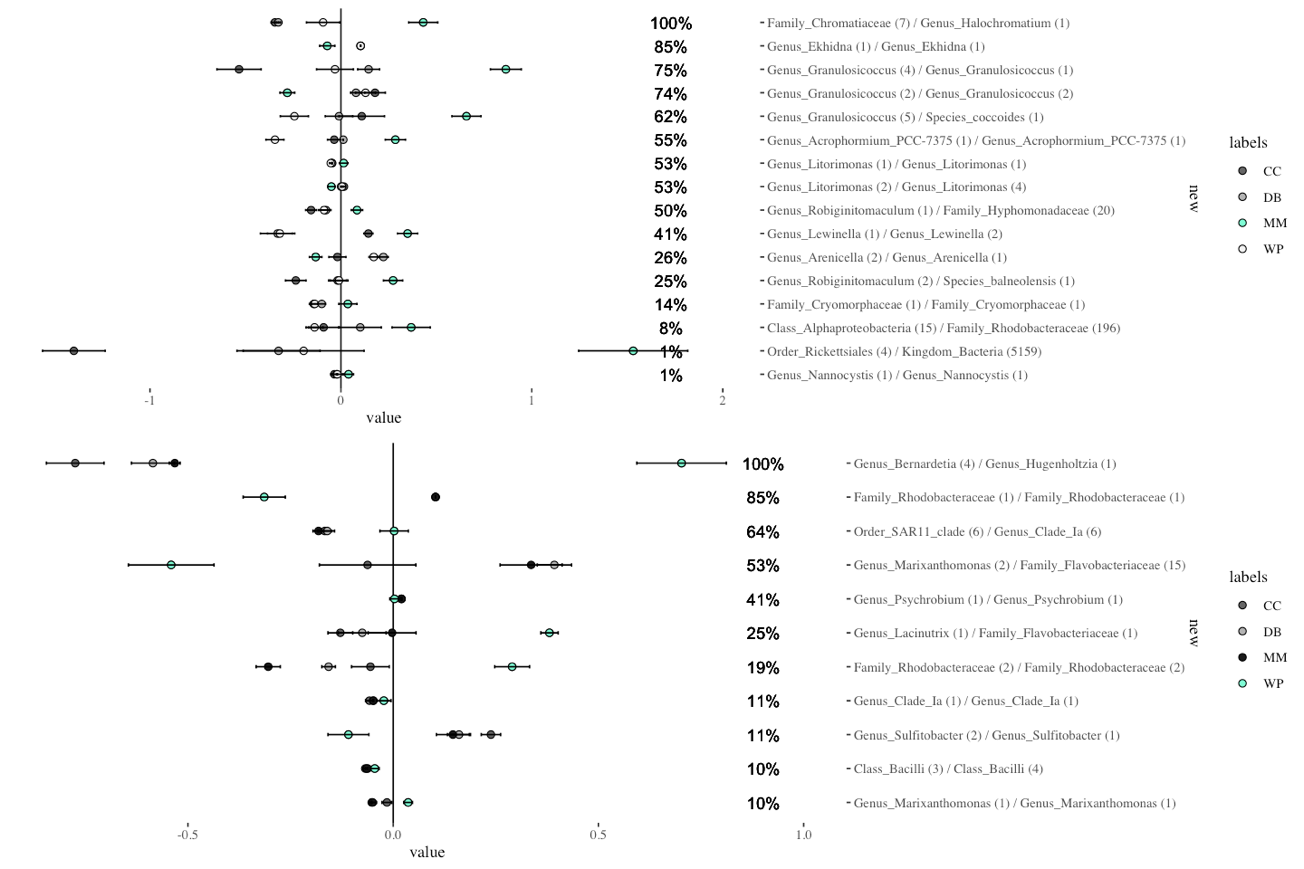

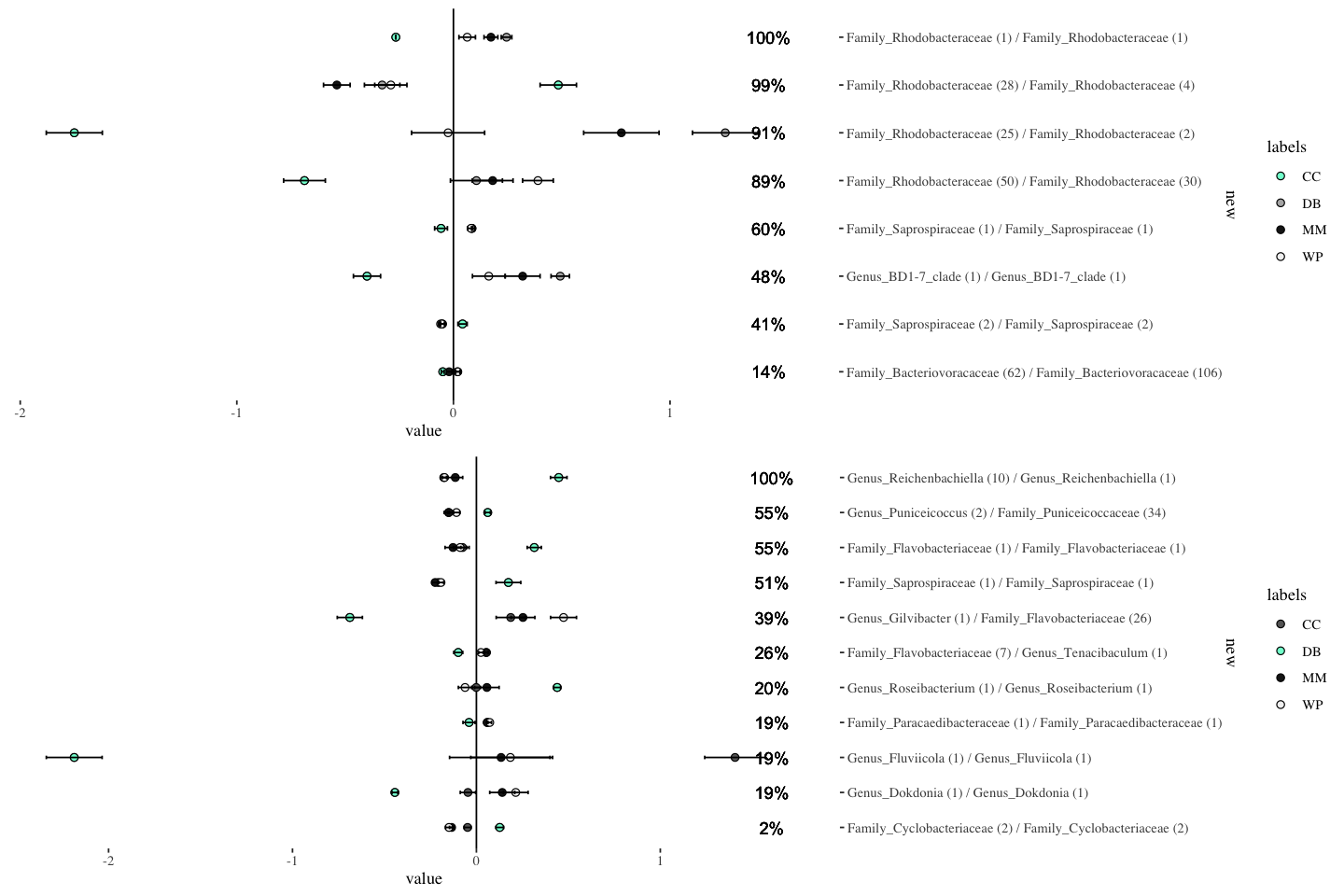
**

Supplemental Figure S5: In roots, after one month, we identified 17 balances distinguishing root bacterial communities at CC, 10 balances at DB, 23 at MM and 20 at WP. See Figure S1 for details on symbols used and interpretation of results.

**
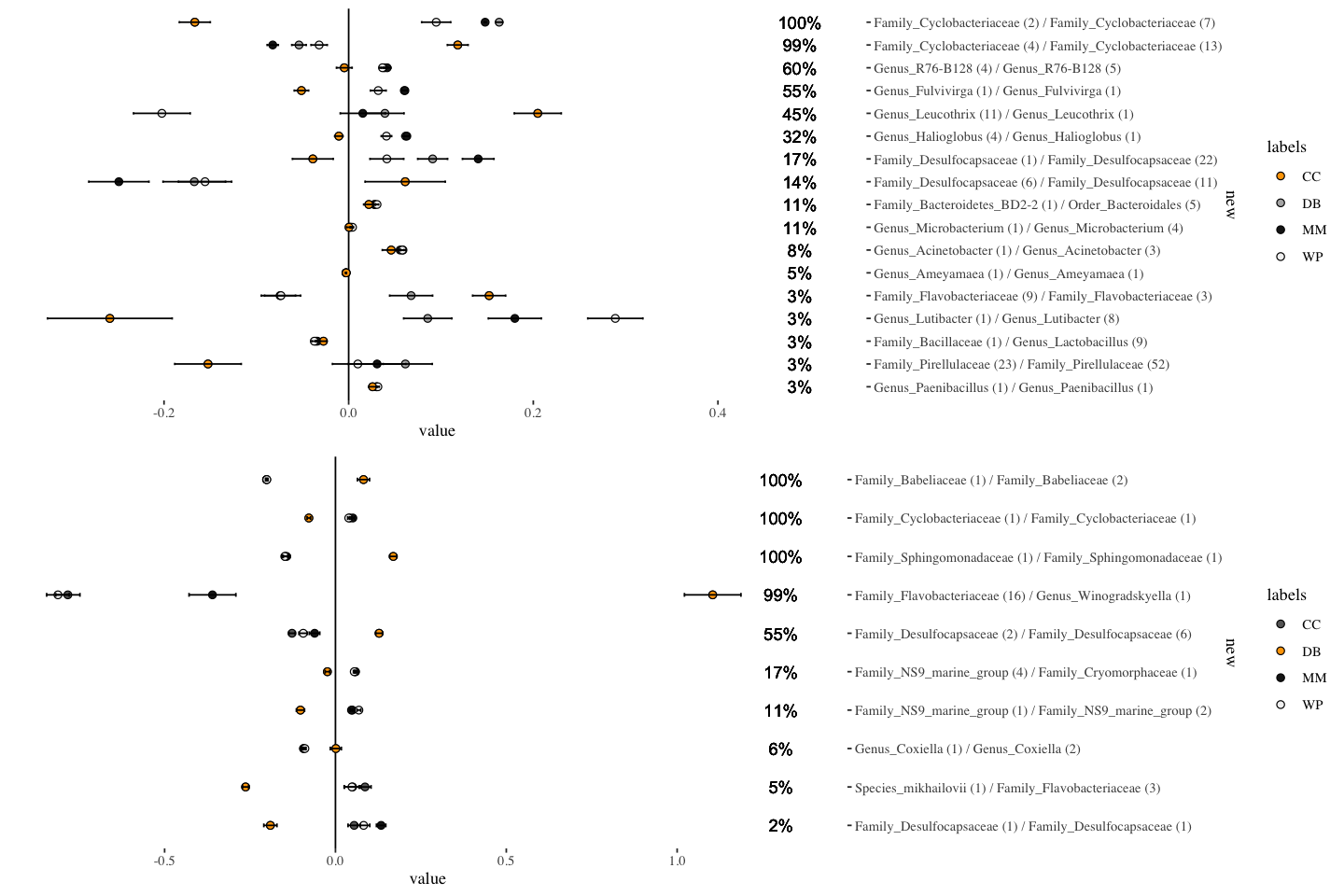

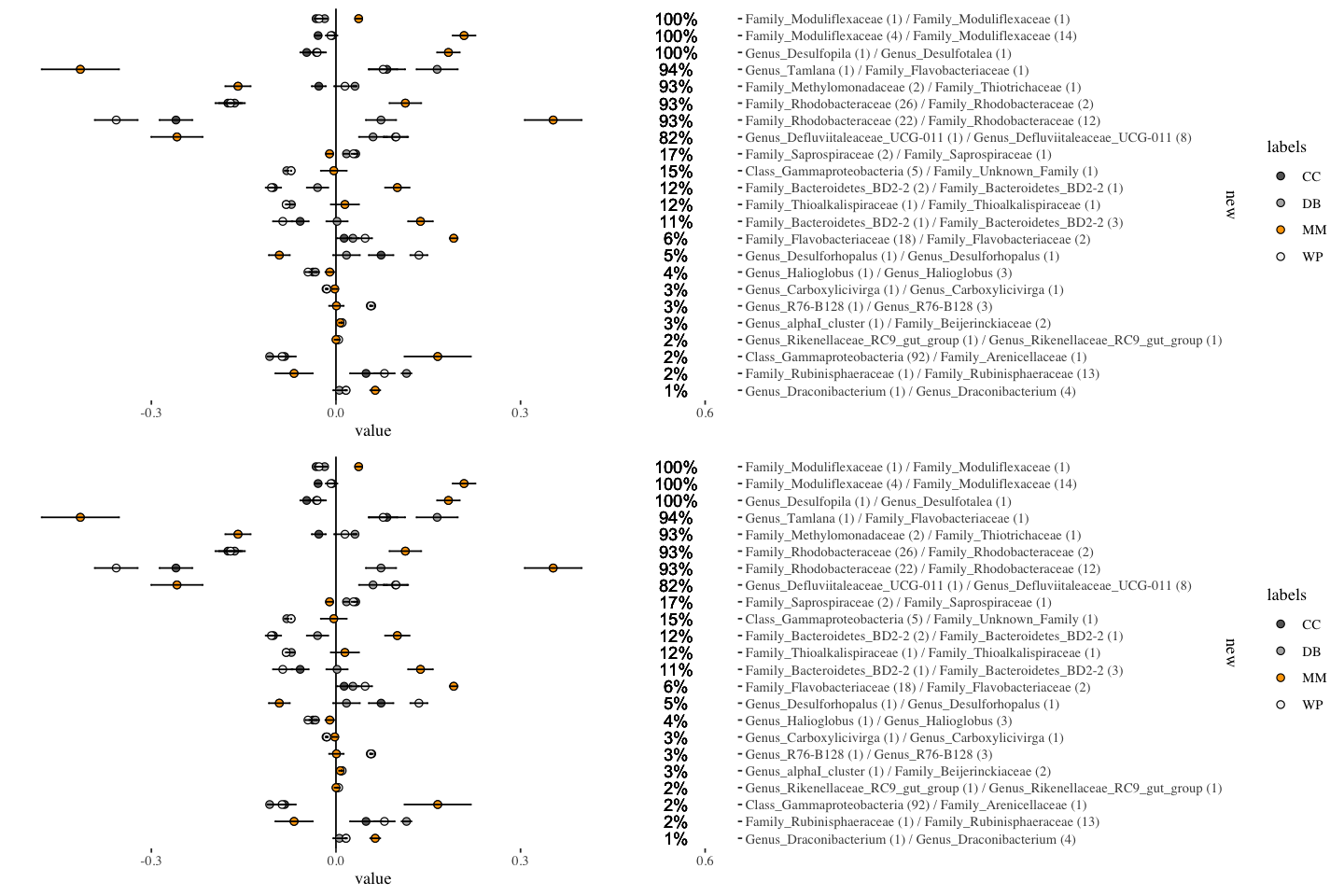
**

Supplemental Figure S6: After two months, we identified 21 balances distinguishing leaf bacterial communities at CC, 10 balances at DB, 18 at MM and 6 at WP.  See Figure S1 for details on symbols used and interpretation of results.

**
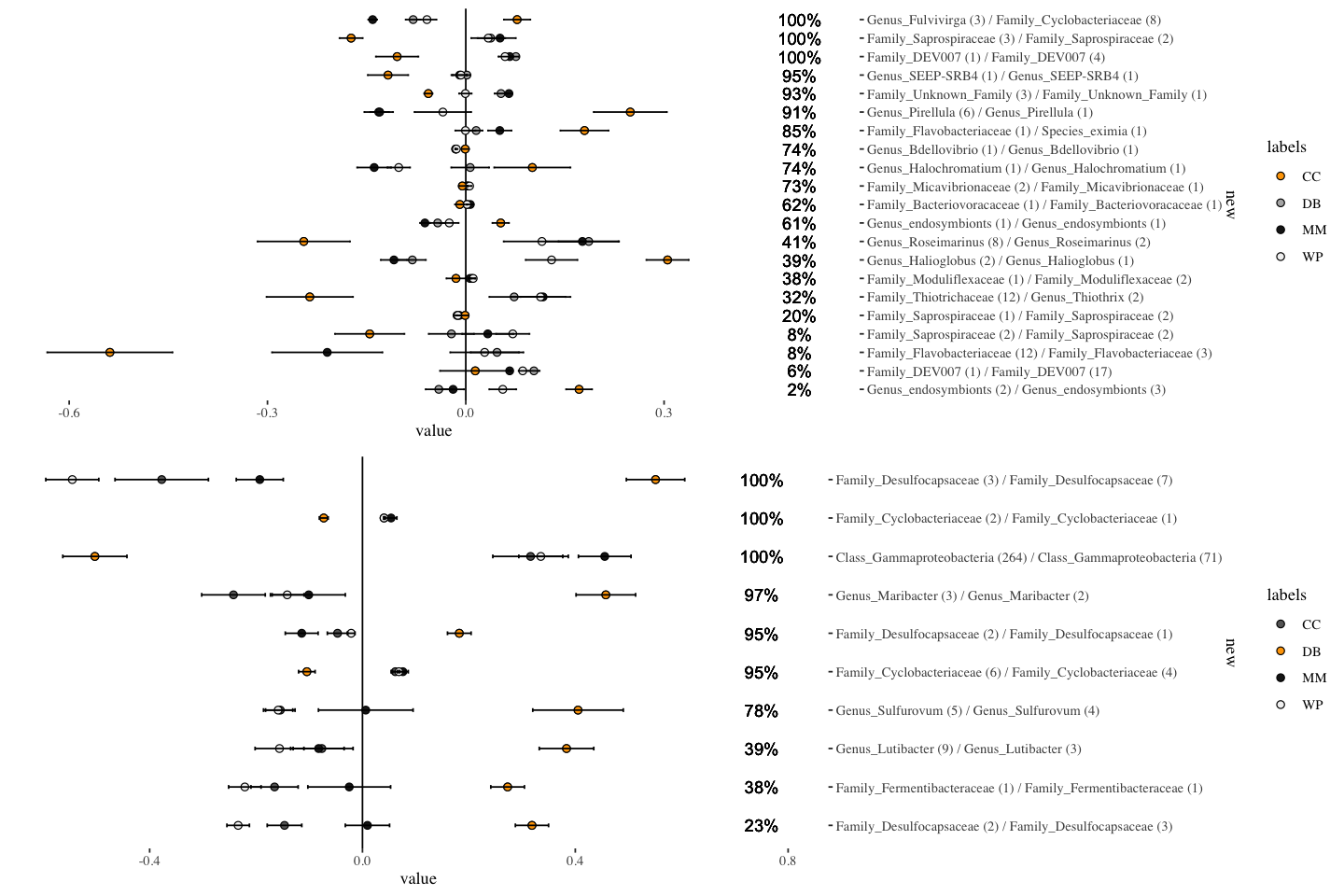

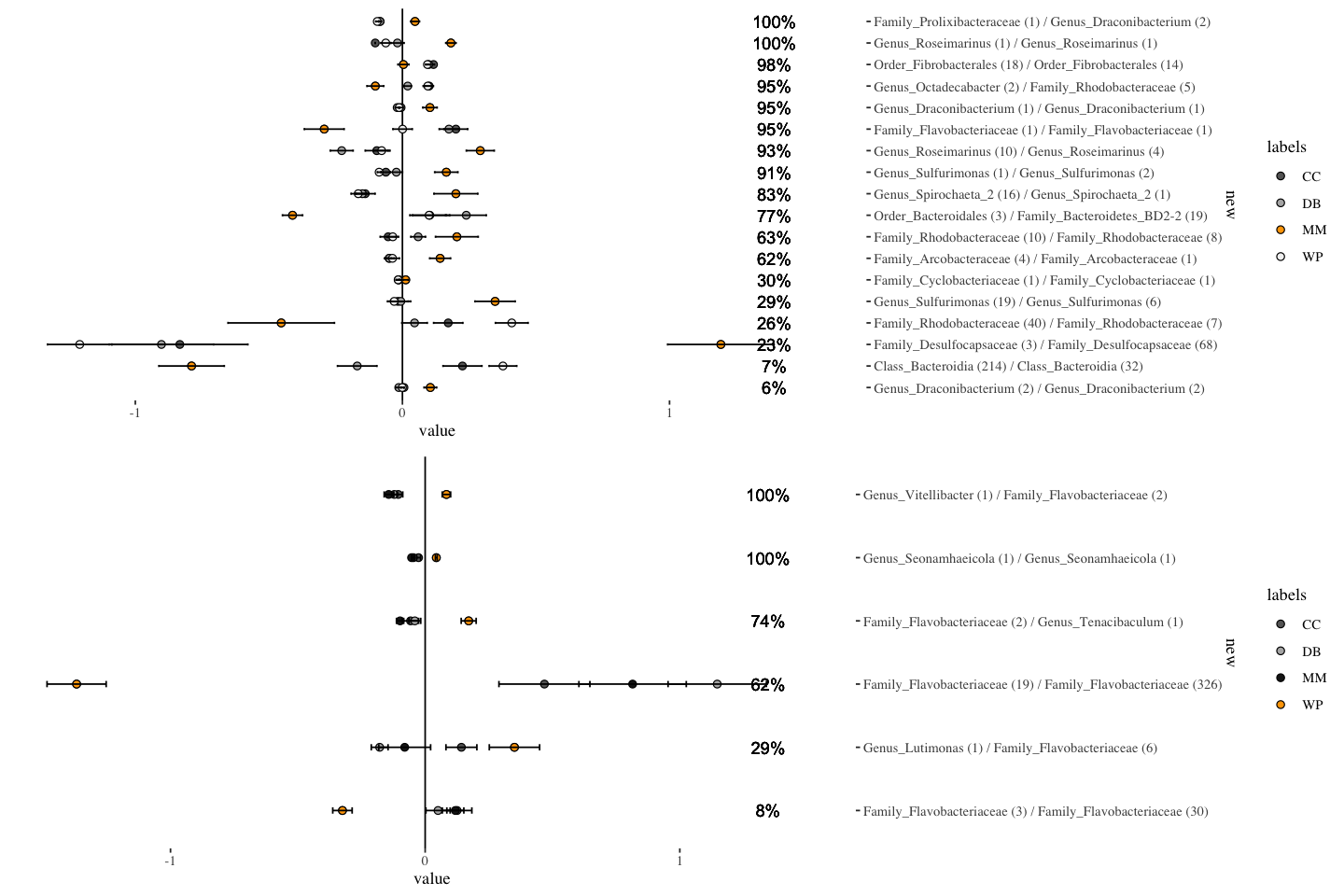
**

Supplemental Figure S7: After three months, we identified 12 balances distinguishing leaf bacterial communities at CC, 5 balances at DB, 15 at MM and 13 at WP. See Figure S1 for details on symbols used and interpretation of results.

**
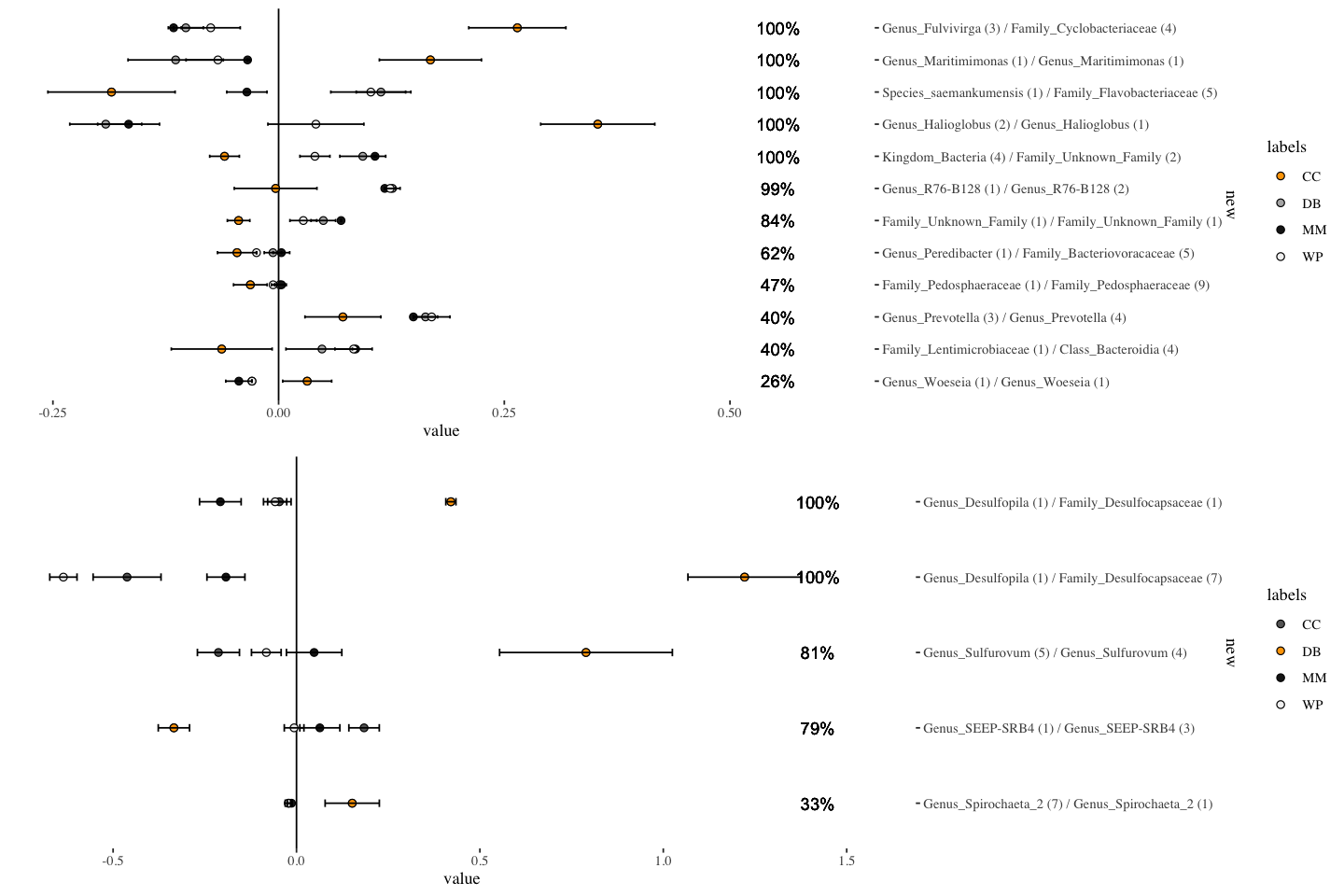

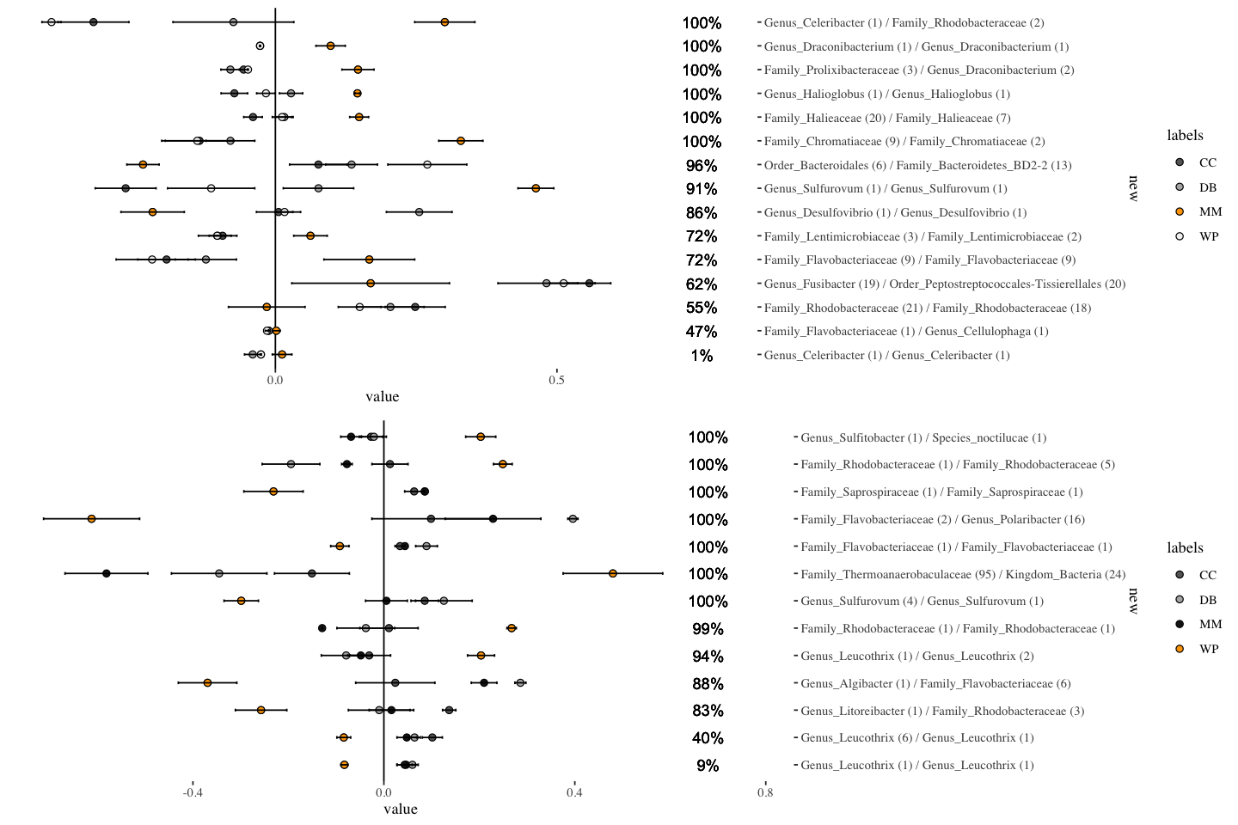
**

Supplemental Figure S8: Mean amplicon sequence variant (ASV) richness on leaves (A-C) and roots (D-F) by transplant status. Here, we plot means and standard errors by time point; the first row is after one month (A, D), the second after two (B, E), and the third after three months (C, F). Only leaves at T3 showed a significant difference in microbial community richness.


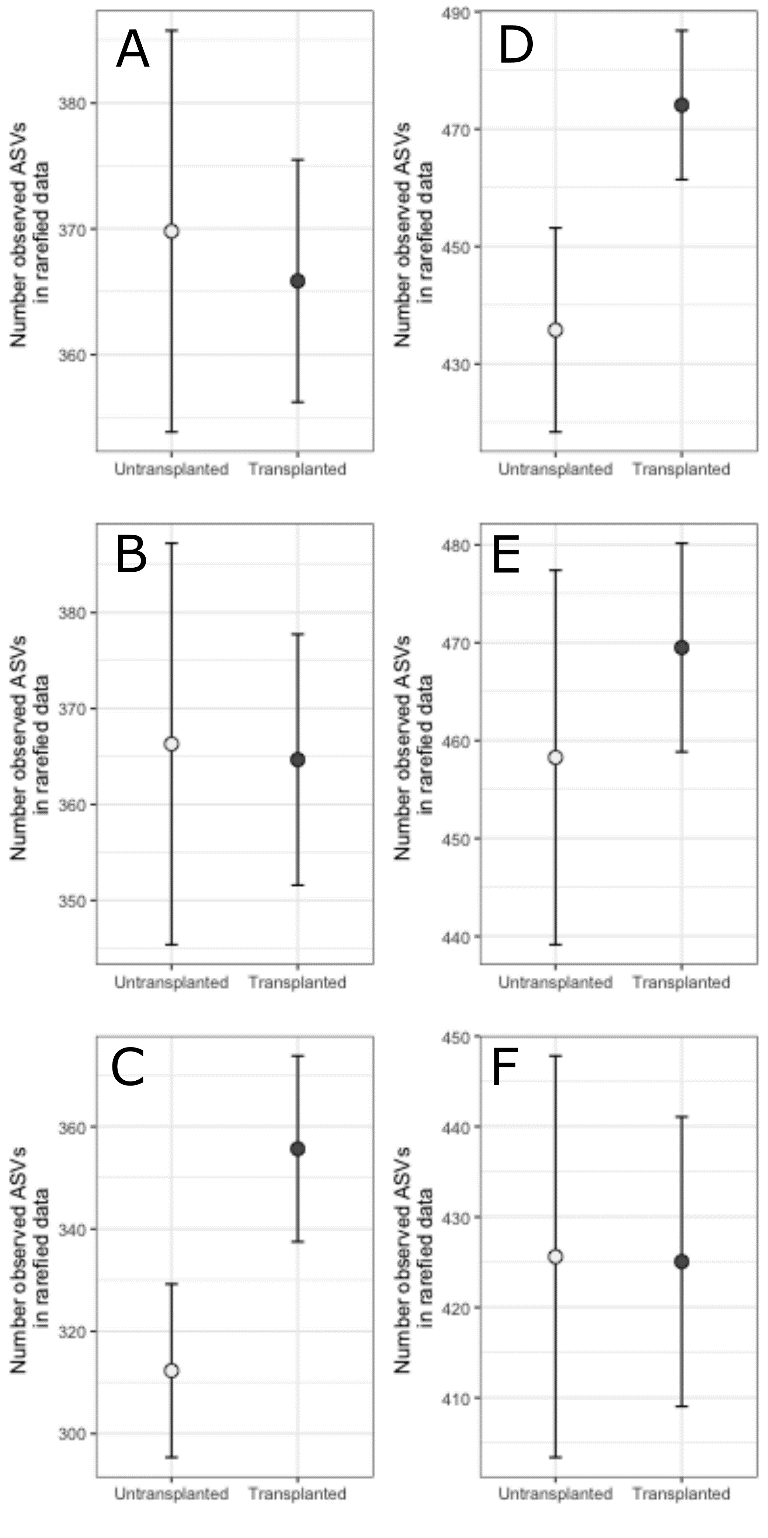


Supplemental Figure S9: Ordination of leaf bacterial community structure based on principal coordinate analysis of phylogenetic-isometric log-ratio transformed distances. Though there is a transplantation effect in root microbiome, this was not due to them changing to resemble leaves. Here brown/yellow points are root communities and green points are leaf communities. Brighter triangles indicate undisturbed controls and darker circles are transplanted plants. While transplanted microbial communities were distinct from undisturbed microbial communities in roots, they still strongly resemble control root microbial communities rather than leaf microbial communities.


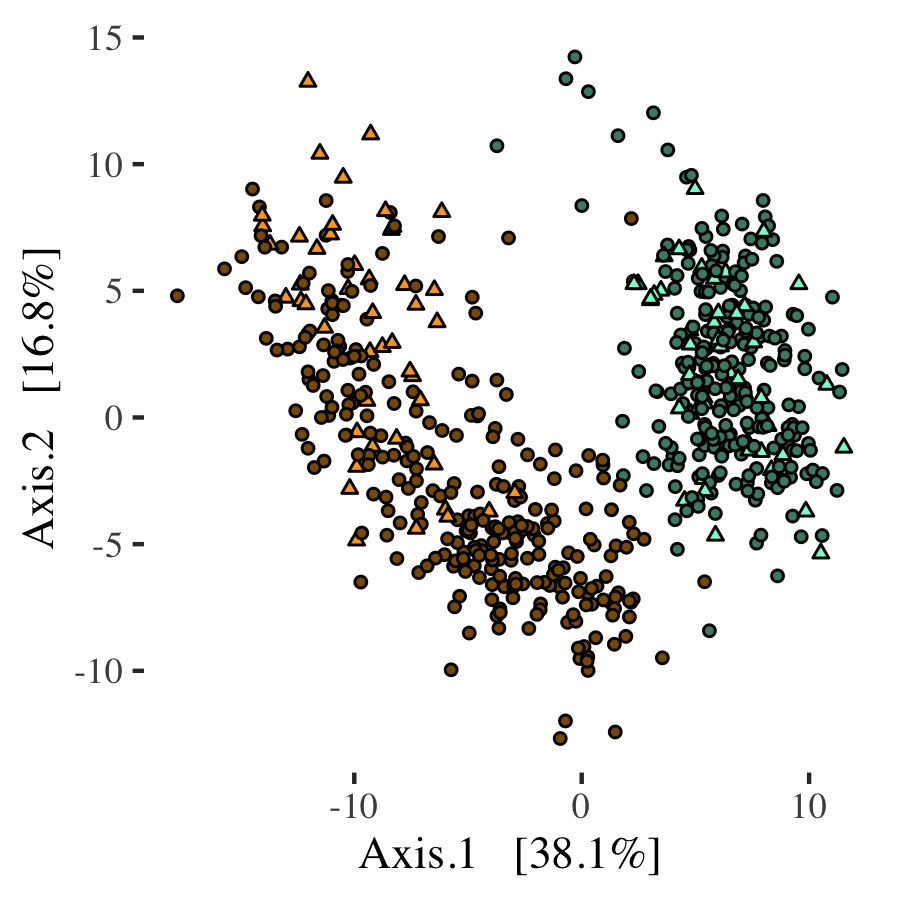

Supplement: Supplementary file 1 — Supplementary Information. [file 41598_2023_30194_MOESM1_ESM.docx]
